# RNA-binding protein SORBS2 suppresses clear cell renal cell carcinoma metastasis by enhancing MTUS1 mRNA stability

**DOI:** 10.1038/s41419-020-03268-1

**Published:** 2020-12-12

**Authors:** Qi Lv, Fan Dong, Yong Zhou, Zhiping Cai, Gangmin Wang

**Affiliations:** 1 Department of Medical Imaging, Tongji Hospital, School of Medicine, Tongji University, Shanghai, China; 2 Department of Urology, Huashan Hospital, Fudan University, Shanghai, China; 3 Department of Urinary, Ningbo Urology and Nephrology Hospital, Ningbo, Zhejiang China; 4 Department of Urology, Changzheng Hospital Affiliated to the Second Military Medical University, Shanghai, China

**Keywords:** Metastasis, Oncogenes, Tumour biomarkers, Cell invasion, Microtubules

## Abstract

RNA-binding proteins (RBPs) predominantly contribute to abnormal posttranscriptional gene modulation and disease progression in cancer. Sorbin and SH3 domain-containing 2 (SORBS2), an RBP, has been reported to be a potent tumor suppressor in several cancer types. Through integrative analysis of clinical specimens, we disclosed that the expression level of SORBS2 was saliently decreased in metastatic tissues and positively correlated with overall survival. We observed that overexpression of SORBS2 brought about decreased metastatic capacity in ccRCC cell lines. Transcriptome-wide analysis revealed that SORBS2 notably increased microtubule-associated tumor-suppressor 1 gene (MTUS1) expression. In-depth mechanistic exploring discovered that the Cys2-His2 zinc finger (C2H2-ZnF) domain of SORBS2 directly bound to the 3′ untranslated region (3′UTR) of MTUS1 mRNA, which increased MTUS1 mRNA stability. In addition, we identified that MTUS1 regulated microtubule dynamics via promoting KIF2C^S192^ phosphorylation by Aurora B. Together, our research identified SORBS2 as a suppressor of ccRCC metastasis by enhancing MTUS1 mRNA stability, providing a novel understanding of RBPs during ccRCC progression.

## Introduction

Clear cell renal cell carcinoma (ccRCC) is the most prevalent histological subtype of kidney cancer and is believed to mainly originate from proximal tubular epithelial cells of the nephron^1^. Genetically, ccRCC is characterized by biallelic loss of Von Hippel-Lindau tumor-suppressor gene that lead to aberrant accumulation of hypoxia-inducible factors (HIF-1α and HIF-2)^2^. Characterized by a lack of early symptoms and conspicuous clinical manifestations, >50% of patients are detected incidentally and 20–30% of patients present with metastasis at the time of diagnosis^3^. Patients with localized ccRCC can be cured by nephrectomy, whereas 30% of them will eventually develop metastases^4^. Despite the arise of tyrosine kinase inhibitors, mammalian target of rapamycin inhibitors, and immune checkpoint inhibitors, patients with metastatic ccRCC often eventually develop drug resistance and succumb to disease with survival time ranging from 3 months to 5 years^5^. Hence, there is an urgent need to elucidate the underlying mechanism of ccRCC metastasis.

Apart from gene mutations that directly involve in cell signaling and repair pathways, many aberrations that take part in the stabilization/destabilization of altered transcriptomic profiles promote the acquisition of metastatic traits^6^. RNA-binding proteins (RBPs) are particularly relevant to transcriptome because they can modulate virtually all steps of post-transcription, from alternative splicing, RNA stability to RNA decay^7^. As a result, RBP malfunction almost influences each step during the development and progression of cancer, including sustaining cell proliferation, resisting apoptosis, avoiding immune surveillance, inducing angiogenesis, and activating invasion and metastasis^8^. Interestingly, examination of sample-wise mRNA–protein correlation indicated a concerted shift of discordant ribosome protein–mRNA levels in ccRCC^9^. Supporting this observation, a systematic interrogation of RBP-mediated regulatory programs has shown that the RBPs contribute significantly to the widespread remodeling of ccRCC transcriptome through regulation of mRNA stability^10^.

Sorbin and SH3 domain-containing protein 2 (SORBS2, also called ArgBP2), a member of the SoHo family of adapter proteins, is previously characterized as adapter protein associated with c-Abl/Arg non-receptor tyrosine kinase pathways and functioned as a regulator of actin-dependent processes, such as cell adhesion and migration^11,12^. SORBS2, which is highly expressed in the normal human tissues, is strongly repressed during the progression and metastasis of cervical carcinogenesis, hepatocellular carcinoma, and pancreatic cancer^13–15^. In addition, SORBS2 has been identified as an RBP to suppress metastasis of hepatocellular carcinoma and ovarian cancer^16,17^. However, the precise role of SORBS2 and the underlying regulatory mechanisms in ccRCC remain poorly understood.

In this study, we identified SORBS2 as a suppressor of ccRCC metastasis. SORBS2 was significantly downregulated in metastatic tissues and positively associated with overall survival and tumor grade. We found that overexpression of SORBS2 can suppress metastasis and epithelial–mesenchymal transition. The C2H2-ZnF domain of SORBS2 directly bound to the 3′ untranslated regions (3′UTRs) of microtubule-associated tumor-suppressor 1 gene (MTUS1) mRNA that promoted microtubule stability. Therefore, our study highlighted SORBS2 as a promising biomarker with diagnostic and therapeutic significance for ccRCC.

## Materials and methods

### Patients and specimens

Ninety-six tumor tissues and their adjacent normal tissues from ccRCC patients who underwent resection without preoperative chemotherapy or radiotherapy were collected from the Department of Urology, Huashan Hospital between March 2017 and May 2019. The primary and metastatic tumor sites were confirmed by radiographic evaluation that consisted of computed tomography, brain magnetic resonance imaging, and bone scans. Informed consent was obtained from all patients and this study was approved by the Ethical Committee of Huashan Hospital. All experiments relevant to clinic specimens were performed in accordance with the principles outlined in the Declaration of Helsinki. All fresh specimens were immediately stored in liquid nitrogen and part of specimens were embedded with paraffin. The clinical characteristics of patients are presented in Table 1.

**Table 1:** Clinicopathological features of patients with ccRCC.

Variables		Total	SORBS2 expression		*P* value
			Low	High	
Sex	Male	36	20	16	0.884
	Female	60	35	25	
Age (years)	<60	43	25	18	0.275
	≧60	56	29	27	
Tumor size (cm)	<4	59	33	26	0.872
	≧4	37	22	15	
Fuhrman grade	I–II	37	0	37	<0.001
	III–IV	59	55	4	
Distant metastasis	Negative	37	0	37	<0.001
	Lymph node	17	15	2	
	Liver	13	11	2	
	Lung	23	23	0	
	Bone	6	6	0	

### Cell line, cell culture, and cell transfection

The human RCC cell lines A-498, 786-O, Caki-2, ACHN, Caki-1, and SKRC45 and human renal tubular epithelial cell line HK2 cells were obtained from American Type Culture Collection (ATCC, Manassas, VA). Caki-1 and 786-O were cultured in McCOY’s 5A (Gibco, China) and RPMI-1640 (Gibco, China), respectively, supplemented with 10% fetal bovine serum (Gibco, Australia source), 100 μg/ml penicillin, and 100 μg/ml streptomycin (Gibco, China) in a humidified atmosphere containing 5% CO_2_ at 37 °C. SORBS2-overexpression cell lines were constructed by infecting with lentivirus containing SORBS2 coding sequence (CDS) and selected the stable cell lines by puromycin. Gene silencing assay was performed using the siRNA-Mate™ transfection reagent (GenePharma, Shanghai, China) according to the manufacturer’s instructions. The small interfering RNAs (siRNAs) were synthesized by GenePharma and the sequences are listed in Table S1.

### Quantitative real-time PCR (qRT-PCR)

Total RNA was extracted using TRIZOL Reagent (Invitrogen) according to the manufacturer’s instructions. cDNA synthesis and PCR amplification was performed using SuperScript IV One-Step RT-PCR System (Takara) according to the manufacturer’s instructions. Glyceraldehyde 3-phosphate dehydrogenase (GAPDH) was used as the internal control. The PCR primers used are listed in Table S1.

### Western blot and co-immunoprecipitation (Co-IP)

Proteins were extracted using the Total Protein Extraction Kit (Solarbio), and protein concentrations were determined using the TaKaRa BCA Protein Assay Kit (TaKaRa) according to the manufacturer’s instructions. Denatured proteins were separated using sodium dodecyl sulfate–polyacrylamide gel electrophoresis, transferred to a polyvinylidene difluoride membrane (Millipore), blocked with skim milk, and then incubated with the indicated primary antibody overnight at 4 °C and probed with secondary antibody at room temperature for 2 h. The primary antibodies were listed as follows: SORBS2 (SAB4200183, MilliporeSigma), E-cadherin (ab1416, Abcam), N-cadherin (ab18203, Abcam), Vimentin (ab92547, Abcam), MTUS1 (ab198176, Abcam), acetylated-tubulin (T6793, MilliporeSigma), β-tubulin (T4026, MilliporeSigma), KIF2C (sc-81305, Santa Cruz Biotechnology), and GAPDH (sc-32233, Santa Cruz Biotechnology). For detection of KIF2C^S192^ phosphorylation (p-S192), autoradiograph was performed. Briefly, wild-type recombinant protein KIF2C^WT^ (1 μg), pointed mutant KIF2C^S192A^ (1 μg), Aurora B (100 ng), and MTUS1 (100 or 500 ng) (recombinant proteins were synthesized and purified by Medicilon, Shanghai) were incubated with 0.2 μCi/μl [γ-^32^P]-ATP (Perkin Elmer, NEG002Z) and 30 μM non-radioactive ATP (Thermo, PV3227) for 30 min at 37 °C. The reaction was terminated by boiling at 95 °C for 10 min. The denatured protein was used for gel electrophoresis. Inasmuch as Co-IP, Caki-1 cell lysate was pulled down by MTUS1 antibody (ab198176, Abcam) at 4 °C for 2 h and then incubated with protein A Sepharose CL-4B beads (GE) at 4 °C overnight. The beads were then washed thrice with RIPA buffer and the precipitates were used for western blot. Protein bands were quantified by the ImageJ software and normalized to internal control GAPDH. The ratio of protein/GAPDH is shown under the protein band.

### Immunohistochemistry staining

Paraffin-embedded tissues were sliced to 4 μm, deparaffinized in xylene, dehydrated in gradient ethanol solutions, rehydrated with phosphate-buffered saline (PBS), incubated with citrate buffer (pH 6.0) for antigen retrieval, blocked by 3% H_2_O_2_, and then incubated with anti-SORBS2 antibody (ab73444, Abcam) overnight at 4 °C. After rinsing thrice with TTBS, sections were incubated with an anti-mouse IgG, horseradish peroxidase-linked antibody (7076, CST) for 30 min at room temperature, followed by visualization using diaminobenzidine (DAB) substrate solution for 30 min and reaction with DAB and counterstaining with Mayer’s hematoxylin.

### Immunofluorescence staining

Caki-1 and Caki-1-SORBS2 (SORBS2-overexpressed Caki-1) cells were grown in 6-well plate for 24 h and then treated with si-MUTS1 or scramble overnight at 37 °C. Cell culture was replaced with 500 nM staining solution (SiR-tubulin Kit, CY-SC002) and incubated in a humidified atmosphere containing 5% CO_2_ at 37 °C. After 3 h, the cells were imaged on a Leica DM5000 fluorescence microscope.

### Wound-healing assay and Transwell assay

Cell migration was assessed by wound healing assay. Cells were grown in 24-well plates. Till they reached 80% confluence, a scratch was made with a 200-μl sterile pipette tip. The wounded closures were imaged at 0 and 24 h after the scratches were made using an inverted microscope. Invasion assay was performed with 24-well BioCoat Matrigel Invasion Chambers (BD) according to the manufacturer’s instructions. The cells that invaded through the Matrigel were first fixed with 4% Paraformaldehyde Fix Solution (Beyotime) for 20 min and then stained with crystal violet (Beyotime) for 15 min at 37 °C. After washing with PBS, 5 randomly selected fields were imaged (×400) and counted.

### RNA immunoprecipitation (RIP) and sequencing (RIP-sequencing)

Cell lysates were incubated with SORBS2 (SAB4200183, MilliporeSigma) overnight at 4 °C and then immunoprecipitated with protein Pierce™ Protein A/G Magnetic Beads. RNA was extracted from the immunoprecipitated SORBS2-RNA complex using Trizol. After proteinase K digestion (Roche) and fragmentation, RNA libraries were constructed by using VAHTS™ Stranded mRNA-seq Library Prep Kit for Illumina (Vazyme) according to the manufacturer’s instructions. After proteinase K digestion (Roche), RNA libraries were constructed by using the TruSeq Stranded Total RNA Library Prep Kit (Illumina, San Diego, CA, USA) according to the manufacturer’s instructions. The library quality was assessed by Agilent 2100 Bioanalyzer. cDNA libraries were sequenced by Illumina Hiseq 4000 system according to the manufacturer’s instructions.

### RNA pull-down assay

The full-length 5′UTR, CDS, and 3′UTR of were MTUS1 synthesized and cloned into pcDNA3.1 plasmid (Sangon Biotech, Shanghai). DNA sequence with an added 5′ T7 RNA polymerase promoter sequence was produced using the primers listed in Table S1. KOD One™ PCR Master Mix (TOYOBO) was used for DNA amplification. The RNA sequence was produced by using T7 RNA Polymerase (Roche). The potential RBPs of MTUS1 mRNA were harvested by using the Pierce™ Magnetic RNA-Protein Pull-Down Kit (Pierce Biotechnology). The binding of SORBS2 to MTUS1 mRNA was further confirmed by western blot. All procedures were performed according to the manufacturer’s instructions.

### RNA stability analysis

To explore the mRNA decay rate of MTUS1 mRNA, SORBS2-overexpression and control cell lines were treated with 5 μg/ml actinomycin D to inhibit RNA transcription. Total RNA was isolated from these cell lines at the indicated time points and the expression of MTUS1 mRNA was analyzed using qRT-PCR. mRNA levels were normalized to the internal control GAPDH and plotted as a percentage of the value at the time of adding actinomycin D.

### Luciferase reporter assay

The full-length 3′UTR and the ∆3′UTR of MTUS1 mRNA was synthesized and cloned into pmirGLO plasmid (Sangon Biotech, Shanghai). The full-length SORBS2 and point mutated SORBS2 was synthesized and cloned into pcDNA3.1 plasmid (Sangon Biotech, Shanghai). For the luciferase reporter assay, cells were seeded into 24-well plate and transfected with the indicated plasmids. After 48 h, the luciferase activity was measured by the Dual-Luciferase Reporter Assay System (Promega).

### Microtubule depolymerization assay

Caki-1 cells were transfected with 2.5 μg plasmid (pcDNA3.1-MTUS1, pcDNA3.1-KIF2C^MT^, pcDNA3.1-KIF2C^S192A^, pcDNA3.1-KIF2C^M^+ pcDNA3.1-MTUS1) using PEIpro (Polyplus). Six hours later, cells were incubated with 1 μM Tubulin Tracker™ Green (Invitrogen) for 30 min and 5% CO_2_. Then the stained cells were analyzed using fluorescence flow cytometry (Beckman). The mean fluorescence intensity was normalized to the control group.

### Statistical analysis

All data were analyzed using the GraphPad Prism 7.0 Software. Student’s *t* test was used to analyze the differences between means of independent groups. The average expression of SORBS was used as a cut-off value for grouping. Kaplan–Meier method was used to evaluate the survival rate. Correlation between SORBS2 and MTUS1 was performed using two-tailed Spearman’s test. All experiments in this study were performed in triplicate. Statistical significance was indicated by *P* values < 0.05. *P < 0.05, **P < 0.01, ***P < 0.001.

## Results

### SORBS2 is downregulated in metastatic ccRCC tissues and positively associated with overall survival

**Fig. 1:**
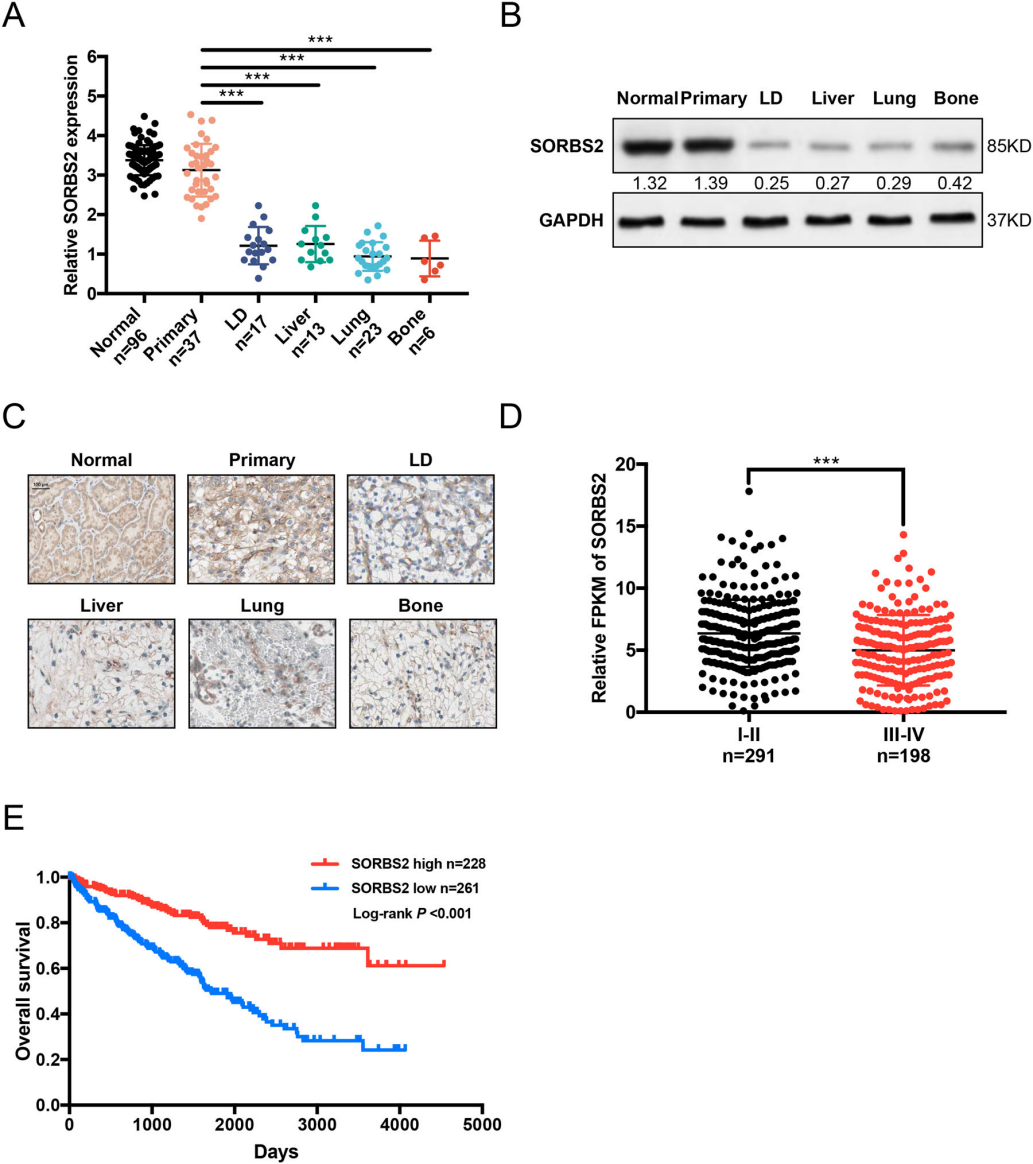
SORBS2 downregulation in ccRCC tissues and its association with poor prognosis. **A** Expression analysis by qRT-PCR of SORBS2 mRNA in different tissues. **B** Expression analysis by western blot of SORBS2 protein in different tissues. **C** Immunohistochemistry analysis of SORBS2 in different tissues, scale bar = 100 μm. **D** Relative FPKM (fragments per kilobase per million) of SORBS2 in different tumor grades. **E** Kaplan–Meier survival curves comparing overall survival in ccRCC patients with high or low SORBS2 expression; the average expression of SORBS was used as a cut-off value for grouping. ***P < 0.001.

To investigate the expression level of SORBS2 in ccRCC, 37 primary tissues, 56 metastatic tissues (including 17 lymph node metastases, 13 liver metastases, 23 lung metastases, and 6 bone metastases), and their corresponding adjacent normal tissues were detected by qRT-PCR. Figure 1A showed that SORBS2 mRNA was dramatically reduced in the metastatic tissues compared to the primary tissues and normal tissues. Consistent with qRT-PCR results, western blot and immunohistochemistry further confirmed significantly lower expression of SORBS2 protein in metastatic tissues than that in primary tissues and normal tissues (Fig. 1B, C). We next obtained 489 ccRCC patients’ datasets from The Cancer Genome Atlas (TCGA). The TCGA data revealed that repressed SORBS2 expression was correlated with higher tumor grade (Fig. 1D and Table S2). Kaplan–Meier survival analysis indicated that patients with low SORBS2 had a poorer overall survival than those belonging to high SORBS2 levels in ccRCC (Fig. 1E). Taken together, these data suggest that SORBS2 might be implicated in metastasis and could be a potential prognostic marker for patients with ccRCC.

### SORBS2 suppresses ccRCC cell metastasis

**Fig. 2:**
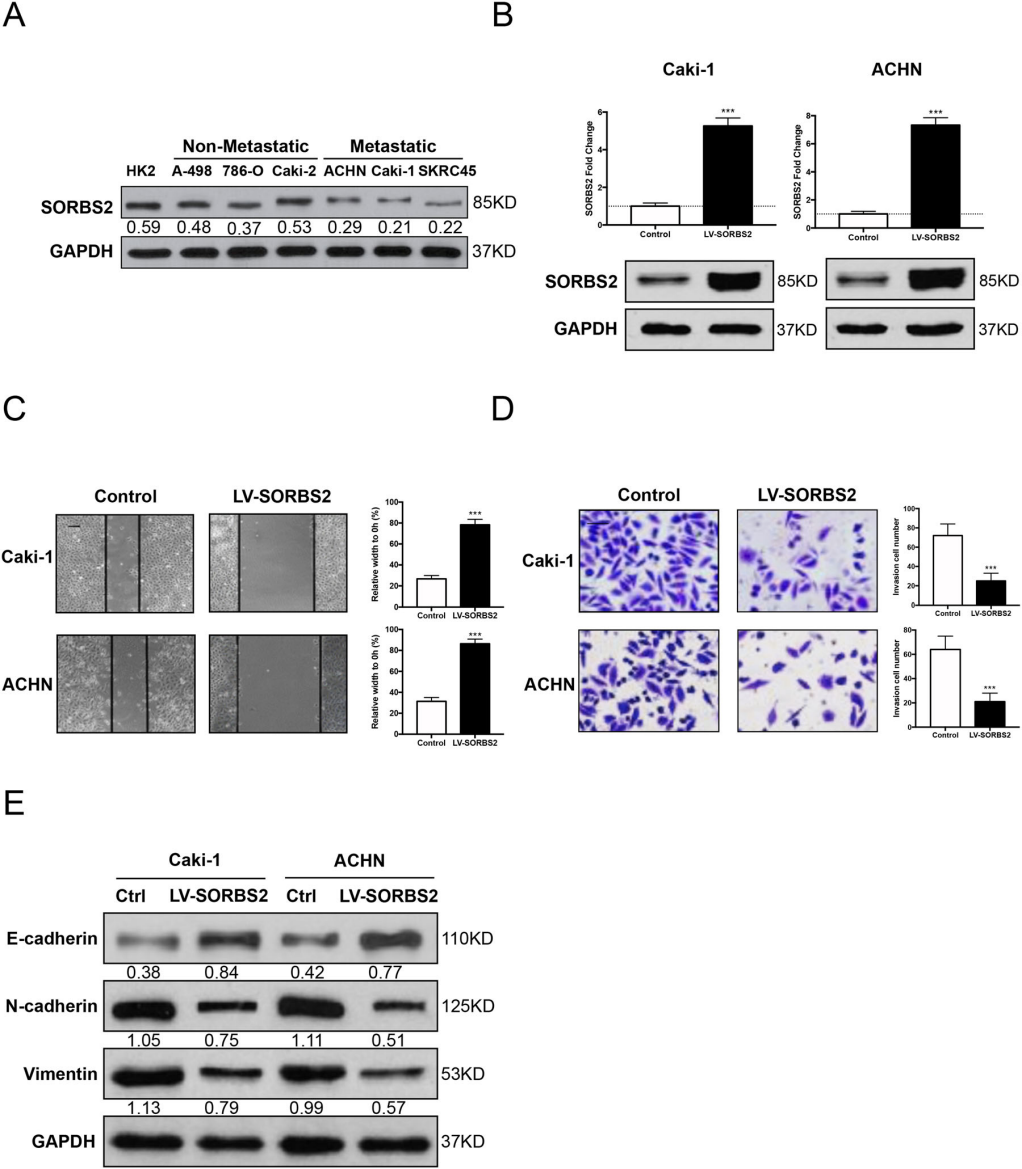
SORBS2 overexpression inhibited ccRCC cell metastasis. **A** Western blot analysis of SORBS2 expression in ccRCC cell lines. **B** Western blot and qRT-PCR analysis of SORBS2-overexpression stable ccRCC cell lines. **C**, **D** Wound healing and Transwell assay, scale bar = 20 μm. **E** Western blot analysis of EMT markers. ***P < 0.001.

To assess the role of SORBS2 in the regulation of ccRCC cell metastasis, we first examined the protein expression of SORBS2 in six different ccRCC cell lines and HK2, a human renal tubular epithelial cell line. Western blot examination showed that SORBS2 was notably lower in highly metastatic cell lines than that in non-metastatic cell lines, which agreed with the clinical observations (Fig. 2A). We then overexpressed SORBS2 in Caki-1 and 786-O cell lines that have low SORBS2 expression using lentivirus containing SORBS2 CDS and selected the stable cell lines by puromycin (Fig. 2B). The wound healing assay showed that SORBS2 overexpression substantially reduced the migration ability of ccRCC cell lines (Fig. 2C). Consistently, the Transwell assay demonstrated that SORBS2 overexpression profoundly decreased the number of cells migrating through the Transwell chamber coated with Matrigel (Fig. 2D). Furthermore, SORBS2 overexpression led to significant upregulation of E-cadherin and downregulation of mesenchymal markers (N-cadherin and Vimentin) (Fig. 2E). Overall, these results indicate that elevated expression of SORBS2 is sufficient to suppress ccRCC metastasis.

### SORBS2 upregulates MTUS1 expression

**Fig. 3:**
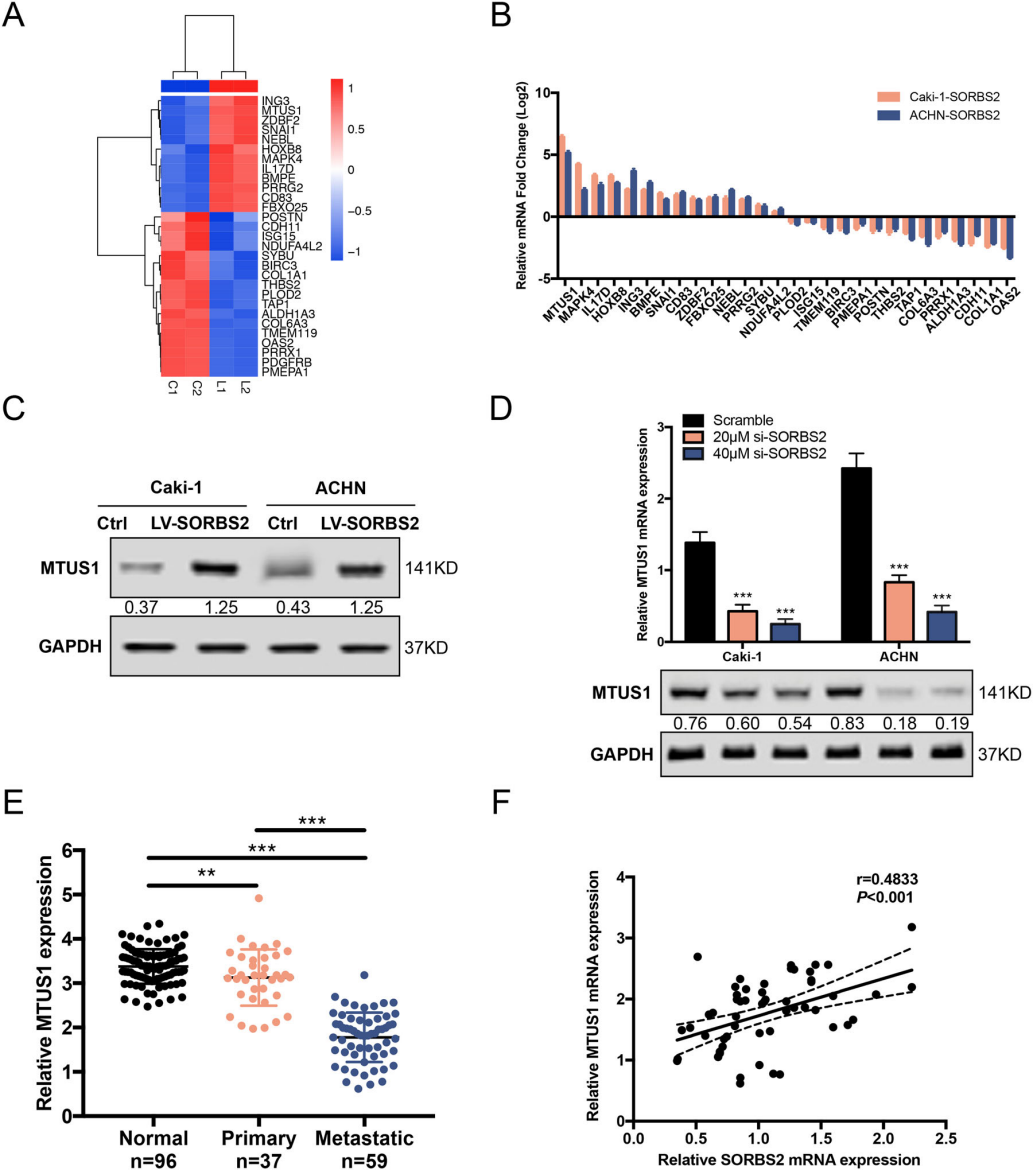
SORBS2 upregulated MTUS1 expression. **A** RIP-sequencing of potential SORBS2-bound genes. **B** qRT-PCR validation of potential SORBS2-bound genes. **C** Western blot analysis of MTUS1 expression in SORBS2-overexpression and control cell lines. **D** Knockdown of SORBS2 led to MTUS1 downregulation. **E** qRT-PCR analysis of MTUS1 expression in ccRCC tissues. **F** Spearman correlation analysis of SORBS2 and MTUS1. **P < 0.01; ***P < 0.001.

To elucidate the underlying molecular mechanisms of SORBS2-meidated metastasis suppression, we performed RIP-sequencing in SORBS2-overexpression cell lines and their corresponding control cell lines. In total, 12 upregulated and 17 downregulated potential SORBS2 targeted genes (fold change ≥2 or ≤−2, adjusted *P* value ≤0.05) (Table S3) were identified in SORBS2-overexpression cell lines vs. control cell lines (Fig. 3A). All these potential SORBS2 targeted genes were further examined by qRT-PCR. qRT-PCR result indicated that MTUS1 was the most significantly changed mRNA upon SORBS2 overexpression (Fig. 3B). Western blot assay further validated upregulation of MTUS1 in SORBS2-overexpression cell lines (Fig. 3C). Subsequently, we repressed SORBS2 expression by siRNA and observed predominantly reduced expression of MTUS1 in accordance with the repression levels of SORBS2 (Fig. 3D). Ultimately, we detected MTUS1 expression in ccRCC patients. Figure 3E revealed a significant decrease of MTUS1 in primary tissues and a drastic reduction in metastatic tissues. In addition, Spearman correlation analysis exhibited a positive correlation between SORBS2 and MTUS1 expression in metastatic tissues (*r* = 0.4833, *P* < 0.001; Fig. 3F). Altogether, these findings indicate that SORBS2 is capable of upregulating MTUS1 expression in ccRCC during the process of metastasis.

### SORBS2 binds to MTUS1 mRNA 3′UTR

**Fig. 4:**
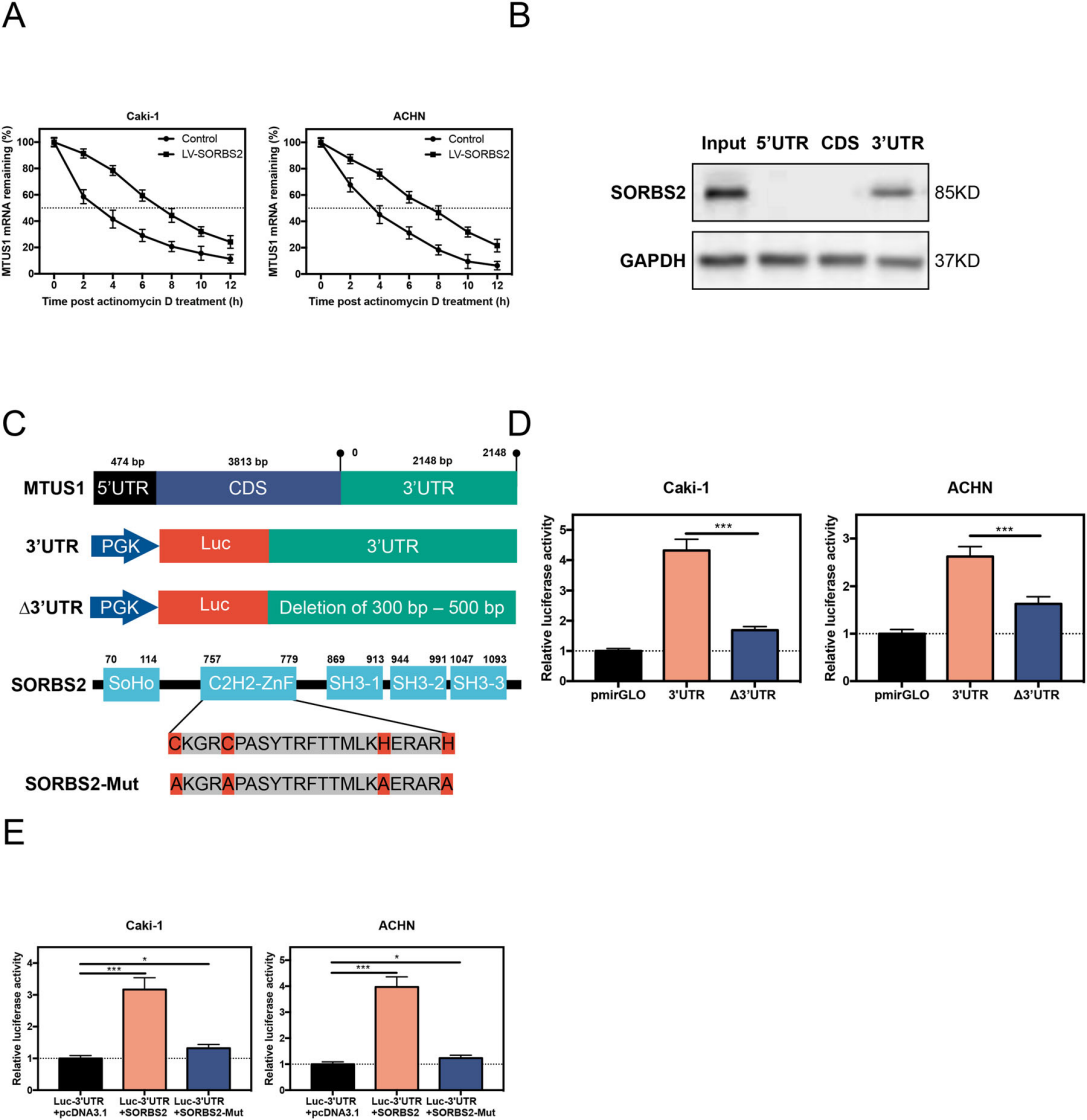
SORBS2 bound to the 3′UTR of MTUS1 mRNA. **A** qRT-PCR analysis of MTUS1 mRNA after treatment with actinomycin D. **B** RNA pull-down analysis of potential binding sequence of SORBS2 to MTUS1 mRNA. **C** Schematic presentations of constructs. **D**, **E** Luciferase reporter assay to determine the binding domain between SORBS2 protein and MTUS1 mRNA. *P < 0.05; ***P < 0.001.

As RBP wield its impact by binding to the target’s mRNA 3′UTR, we next explored the mRNA decay rate of MTUS1 mRNA. Figure 4A showed that overexpression of SORBS2 significantly extended MTUS1 mRNA stability when treated with actinomycin D, an RNA polymerase inhibitor. RNA pull-down directly confirmed that SORBS2 bound to the 3′UTR of MTUS1 mRNA (Fig. 4B). To dissect the detailed interaction between SORBS2 and MTUS1 mRNA 3′UTR, we used catRAPID to predicate the binding domain^18^. catRAPID predicates high binding propensity between the 300th and 500th base pair (bp) of MTUS1 mRNA and nearly the 700th amino acid (aa) of SORBS2 (Fig. S1). For better understanding, a number of schematic presentations are listed in Fig. 4C. Dual-luciferase reporter assay was performed for experiment validation. We observed that deletion of the 300–500 bp of MTUS1 mRNA 3′UTR significantly decreased the luciferase activity (Fig. 4D). We noticed that the 700th aa of SORBS2 was part of zinc finger domain. Thus we substituted the four most critical aa (two Cys and two His) with Ala. Figure 4E demonstrated that transfection of full-length SORBS2 notably increased luciferase activity, whereas transfection of mutated SORBS2 profoundly reduced luciferase activity. Collectively, these data indicate that the C2H2-ZnF domain of SORBS2 directly bound to the 3′UTR of MTUS1 mRNA.

### Knockdown of MTUS1 attenuates SORBS2-mediated metastasis suppression

**Fig. 5:**
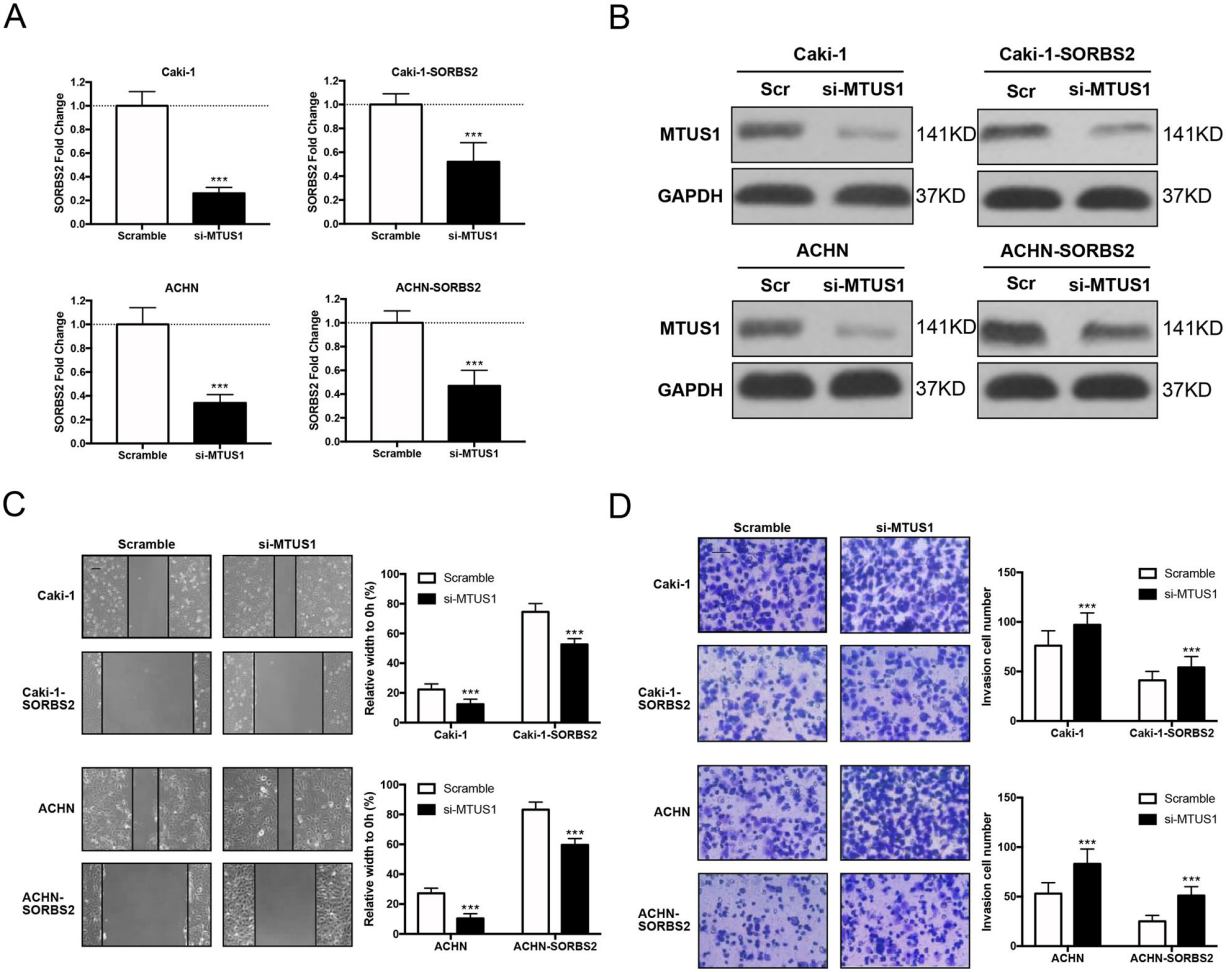
Knockdown of MTUS1 attenuated SORBS2-mediated metastasis suppression. **A**, **B** qRT-PCR and western blot analysis of MTUS1 knockdown in ccRCC cell lines. **C**, **D** Wound healing and Transwell assay, scale bar = 20 μm. ***P < 0.001.

Given that MTUS1 was a critical downstream effector of SORBS2, we attempted to characterize the functional role of MTUS1 in ccRCC metastasis. We first repressed MTUS1 in SORBS2-overexpression cell lines and control cell lines. Figure 5A, B indicated significant knockdown of MTUS1 by si-MTUS1 in mRNA and protein levels. Next, migration assay and Transwell assay were performed to evaluate the functional role of MTUS1 in ccRCC metastasis. Figure 5C, D demonstrated that MTUS1 knockdown dramatically increased the migratory and invasive ability of Caki-1 and ACHN cell lines and partially alleviated SORBS2-overexpression-induced metastasis suppression.

### MTUS1 regulates microtubule dynamics via promoting KIF2C^S192^ phosphorylation by Aurora B

**Fig. 6:**
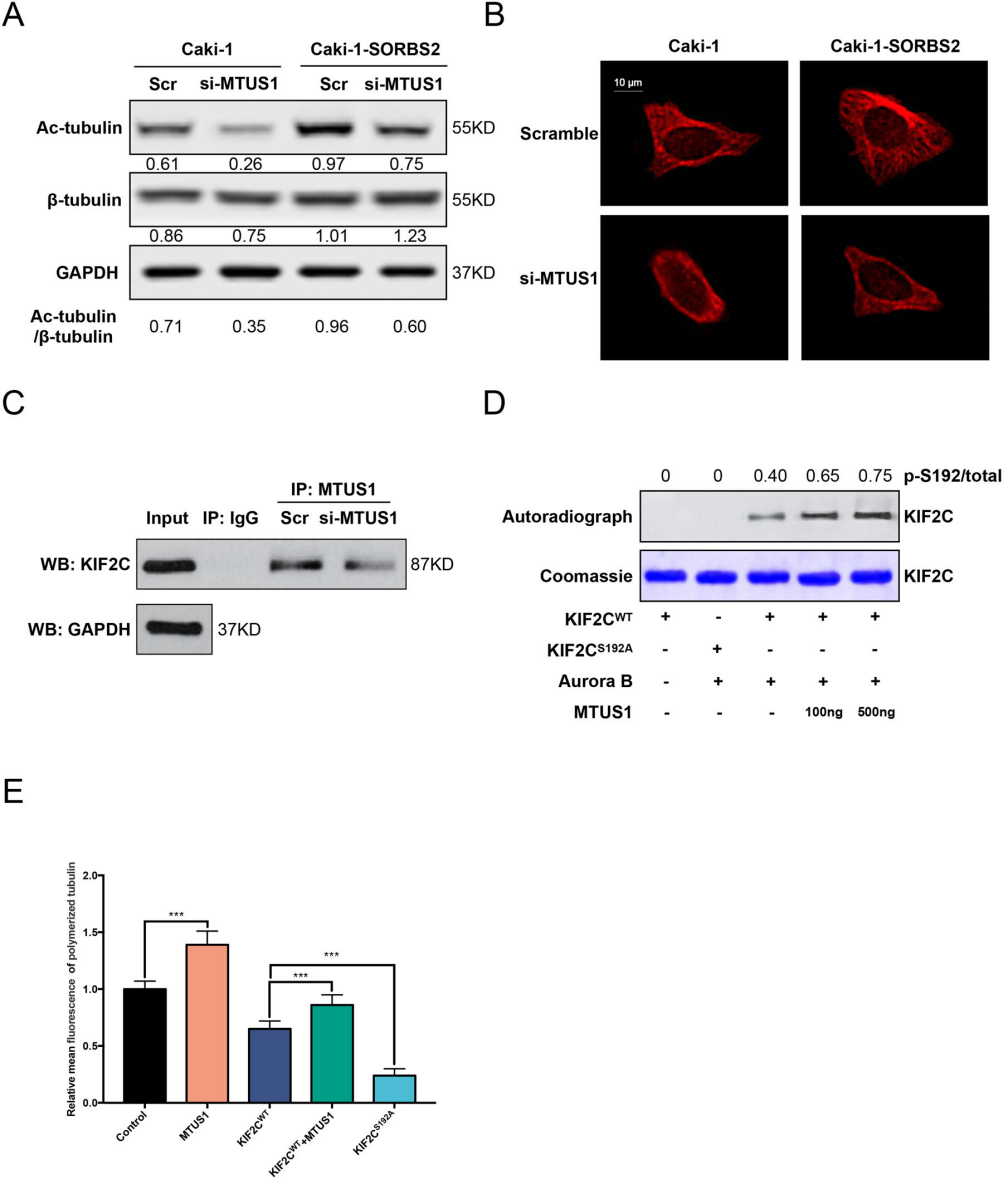
MTUS1 promoted KIF2C^S192^ phosphorylation by Aurora B to regulate microtubule dynamics. **A** Western blot analysis of acetylated-tubulin (Ac-tubulin) when MTUS1 was repressed. **B** Immunofluorescence analysis of tubulin when MTUS1 was repressed, scale bar = 10 μm. **C** Co-IP assays was performed to determine the binding of MTUS1 to KIF2C. **D** Autoradiograph was performed to determine KIF2C^S192^ phosphorylation. **E** Fluorescence flow cytometry was performed to determine the polymerized tubulin. ***P < 0.001.

Note that MTUS1 is a potent microtubule-stabilizing protein^19^, so we assessed the impact of MTUS1 knockdown on microtubule dynamics. We detected a marked decrease of acetylated tubulin, a marker of stabilized microtubules^20^, upon MTUS1 knockdown (Fig. 6A). Immunofluorescent staining of tubulin demonstrated that MTUS1 knockdown led to reduced microtubule extension and halo-like distribution of microtubule that indicated destabilized microtubule network (Fig. 6B). To explore the direct molecule-linked microtubule dynamics and MTUS1, STING database^21^ was utilized to predicate the potential molecules (Fig. S2). We focused on KIF2C (Kinesin family member 2C, also called MCAK) because this gene is significantly associated with microtubule destabilization^22^. Co-IP assays confirmed the direct binding of MTUS1 to KIF2C (Fig. 6C). Previous study identified that the depolymerase activity of KIF2C was regulated by Aurora B kinase phosphorylation^23^. We then examined the phosphorylation status of KIF2C. Autoradiograph image indicated that Aurora B kinase mainly phosphorylated the S192 site of KIF2C (Fig. 6D, lanes 2 and 3), which was consistent with the review by Ritter et al.^24^. Figure 6D (lanes 3–5) revealed that MTUS1 promoted KIF2C^S192^ phosphorylation by Aurora B in a dose-dependent manner. Phosphorylation of S192 site could lead to the inhibition of KIF2C depolymerase activity and reduced directional migration and invasion of tumor cells^25^. To quantify the impact of MTUS1 on KIF2C depolymerase activity, the polymerized tubulin was assessed by fluorescence flow cytometry. In Fig. 6E, MTUS1 notably promoted the formation of polymerized tubulin and partially abolished KIF2C^WT^-induced microtubule depolymerization. In all, these results suggest that MTUS1 regulates microtubule dynamics via promoting KIF2C^S192^ phosphorylation by Aurora B.

## Discussion

Binding of RBPs to regulatory regions (*cis* elements) in the UTRs of mRNAs is a pivotal posttranscriptional regulation during cancer development and metastatic progression^26^. A group of RBPs that regulate the UTRs of mRNAs are implicated in RCC progression and metastasis^27–30^. Here we identified the RBP SORBS2 as a tumor suppressor in ccRCC. Loss of SORBS2 reduced the mRNA stability of MTUS1, which could lead to microtubule destabilization in ccRCC cells. These results highlighted the significance of posttranscriptional regulation and the importance of RBPs in modulating ccRCC metastasis.

SORBS2 is an adapter protein containing three types of domain: N-terminal SoHo domain, three C-terminal SH3 domains, and intermediate C2H2-ZnF domain. The SoHo domain is implicated in binding to the lipid raft protein flotillin and the three SH3 domains are known to interact with poly-proline motifs of c-Arg and c-Abl kinases^31,32^. To our knowledge, Zhao et al. reported that the C2H2-ZnF domain of SORBS2 recognized and stabilized WFDC1 and interleukin-17D mRNA^17^. C2H2-ZnF domain is characterized by the coordinated binding of a zinc ion with two conserved cysteine (Cys) and histidine (His) residues^33^. C2H2-ZnF proteins comprise a multiple family of DNA-binding proteins and RBPs^34^. They are supposed to regulate the expression of downstream genes by modulating the interaction between DNA/RNA sequences and C2H2-ZnF motif^35^. Our investigation on SORBS2 supported the RNA-binding property of the C2H2-ZnF domain from SORBS2.

Metastasis is the most lethal attribute of neoplasms and gives rise to >90% of all cancer-related deaths^36^. Strikingly, ccRCC takes up >85% of metastatic RCC^37^. The most common spreading sites of metastases were lung (45%), bone (30%), lymph node (22%), liver (20%), adrenal (9%), and brain (8%)^38^. Metastasis is a complex and dynamic process that requires the dissemination of cancer cells from primary tumor cells to colonization at distant organs^39^. Dissemination of cancer cells is an intricate cell motility phenomenon that requires dramatic reorganization of the cell cytoskeleton and the concomitant formation of F-actin-rich membrane protrusions known as invadopodia^40–42^. Microtubules, build of α, β-tubulin heterodimers, are fundamental components of the cytoskeleton providing the driving force for cell migration^43^. The balance between microtubule dynamics and stability is of key importance for cell migration^44^. Upregulation of acetylated microtubules, a marker of long-lived and stabilized microtubules, otherwise may be an indicator of repressed microtubule dynamics. Stabilization of microtubules by paclitaxel inhibited colorectal cancer cell migration and invasion in vitro and tumor metastasis in vivo^45^. Moreover, the elongation of invadopodia required the fast dynamic construction of microtubules^46^.

MTUS1 is localized at chromosome 8p22 and comprises 17 exons^47^. Downregulation or loss of MTUS1 is a frequent event and indicator of poor survival in bladder carcinomas, lung cancer, salivary adenoid cystic carcinoma, gastric cancer, and breast cancer^48–52^. In addition, depletion of MTUS1 increases microtubule dynamics and contributes to metastasis in breast cancer^19^. Consistent with our observation, MTUS1 was significantly downregulated in ccRCC tissues, especially in metastatic tissues. Knockdown of MTUS1 decreased microtubule stability, whereas increased microtubule dynamics by promoting the ratio of unphosphorylated KIF2C^S192^ adapting to high motility of metastatic cancer cells.

In summary, our data identified a tumor-suppressor role of SOBRBS2 and unveiled that SOBRBS2, as an RBP, upregulated downstream target MTUS1 expression by binding to its mRNA 3′UTR. Our study highlighted SORBS2 as a promising biomarker with diagnostic and therapeutic significance for ccRCC.

## Supplementary information

Table S1

Table S2

Table S3

Figure S1

Figure S2

Supplementary Figure Legends

## Data Availability

All data that support the findings of this study are available on request from the corresponding author. The data are not publicly available because of privacy or ethical restrictions.
