# Correction to: RNA-binding protein SORBS2 suppresses clear cell renal cell carcinoma metastasis by enhancing MTUS1 mRNA stability

**DOI:** 10.1038/s41419-021-03496-z

**Published:** 2021-11-08

**Authors:** Qi Lv, Fan Dong, Yong Zhou, Zhiping Cai, Gangmin Wang

**Affiliations:** 1 Department of Medical Imaging, Tongji Hospital, School of Medicine, Tongji University, Shanghai, China; 2 Department of Urology, Huashan Hospital, Fudan University, Shanghai, China; 3 Department of Urinary, Ningbo Urology and Nephrology Hospital, Ningbo, Zhejiang China; 4 Department of Urology, Changzheng Hospital Affiliated to the Second Military Medical University, Shanghai, China

**Keywords:** Metastasis, Oncogenes, Tumour biomarkers, Cell invasion, Microtubules

Correction to: *Cell Death and Disease*

10.1038/s41419-020-03268-1, published online 12 December 2020

The original version of this article unfortunately contained a mistake. The authors found a data error in Table 1 about the number of tumor size. The number of tumor size ≧4 should have been 37. The number of high SORBS2 expression in tumor size ≧4 group should have been 15. The corrected Table 1 can be found below. The authors apologize for the mistake. The original article has been corrected.

**Table 1:** Clinicopathological features of patients with ccRCC.

Variables		Total	SORBS2 expression		*P* Value
			Low	High	
Sex	Male	36	20	16	0.884
	Female	60	35	25	
Age (year)	<60	43	25	18	0.275
	≧60	56	29	27	
Tumor size (cm)	<4	59	33	26	0.872
	≧4	37	22	15	
Fuhrman grade	I–II	37	0	37	<0.001
	III–IV	59	55	4	
Distant metastasis	Negative	37	0	37	<0.001
	Lymph node	17	15	2	
	Liver	13	11	2	
	Lung	23	23	0	
	Bone	6	6	0	

