# Supplementary material for: RNA-binding protein SORBS2 suppresses clear cell renal cell carcinoma metastasis by enhancing MTUS1 mRNA stability
Source: Cell Death Dis. 2020 Dec 12;11(12):1056. doi: 10.1038/s41419-020-03268-1 (PMC7732854; doi:10.1038/s41419-020-03268-1)
Supplement: Supplementary file 1 — Table S1 [file 41419_2020_3268_MOESM1_ESM.docx]

**Table S1. The sequences of primers and siRNA**

| **Name** | **Sequence** |
| --- | --- |
| ING3-F | CAGCTTCCTATGGATCTGCGG |
| ING3-R | ATCTCGCGCATTTCCGTGA |
| MTUS1-F | CCGGGGAGAGCTAGTCACT |
| MTUS1-R | CTGCTGGACGAATGCTTCA |
| ZDBF2-F | CAAGGATATTGCAGTTATTGCCG |
| ZDBF2-R | AGACGATGACCCAGTATTCACAT |
| SNAI1-F | TCGGAAGCCTAACTACAGCGA |
| SNAI1-R | AGATGAGCATTGGCAGCGAG |
| NEBL-F | AGAGGCTTTACTCCCGTCGT |
| NEBL-R | ACCCCTTTATAGGCAGCATCG |
| HOXB8-F | GTCCCTGCGCCCCAATTATTA |
| HOXB8-R | GCCCGTGGTAGAACTCCTG |
| MAPK4-F | TGAGAAGGGTGACTGCATCG |
| MAPK4-R | ACCAAACCATTGACACCGAAG |
| IL17D-F | GCCCTGGGCCTACAGAATC |
| IL17D-R | CGCCCTGTTTGTCGATGCT |
| PRRG2-F | AGTAGCCATACCCGGATTCCA |
| PRRG2-R | ACACCTCTCTTCCAGACACTC |
| CD83-F | GAGAAACCTAAGTGGCAAGGTG |
| CD83-R | AGGACAATCTCCGCTCTGTAT |
| BMPE-F | AGCACCTTAGTCACATACCCC |
| BMPE-R | GCTGTGCAGTTATCGTACAGAA |
| FBXO25-F | TCTGCAAGACCTAAGCTCTACC |
| FBXO25-R | TTGCCAGGCGAGAATAGTTTC |
| SORBS2-F | AAGCACAGCCTGCAAGACCA |
| SORBS2-R | TGGGGTATTGGAGGGTCAGG |
| POSTN-F | CTCATAGTCGTATCAGGGGTCG |
| POSTN-R | ACACAGTCGTTTTCTGTCCAC |
| CDH11-F | GTATCCTCGAAGGACAACCCT |
| CDH11-R | GACATCGGTCAGTGTGATCGT |
| ISG15-F | CGCAGATCACCCAGAAGATCG |
| ISG15-R | TTCGTCGCATTTGTCCACCA |
| NDUFA4L2-F | ATGATCGGCTTAATCTGCCTG |
| NDUFA4L2-R | TCCGGGTTGTTCTTTCTGTCC |
| SYBU-F | AGCGCCGACTCCATGAAAG |
| SYBU-R | CTCTACCCGGTGACACTCCTC |
| BIRC3-F | GCCATTGACTTTTCTGTCGCC |
| BIRC3-R | GCAAAGCAAGCCACTCTGT |
| COL1A1-F | GAGGGCCAAGACGAAGACATC |
| COL1A1-R | CAGATCACGTCATCGCACAAC |
| THBS2-F | GACACGCTGGATCTCACCTAC |
| THBS2-R | GAAGCTGTCTATGAGGTCGCA |
| PLOD2-F | GAAAGACACTCCGATCAGAGATG |
| PLOD2-R | CGGCAAAGAGCCATATCAGGA |
| TAP1-F | CTGGGGAAGTCACCCTACC |
| TAP1-R | CAGAGGCTCCCGAGTTTGTG |
| ALDH1A3-F | CCCTGGAGACGATGGATACAG |
| ALDH1A3-R | TCTGAGGGTTCTAATACAGCCC |
| COL6A3-F | CTGTTCCTCTTTGACGGCTCA |
| COL6A3-R | CCTTGACATCATCGCTGTACTGA |
| TMEM119-F | CGGCCTATTACCCATCGTCC |
| TMEM119-R | CTGGGCTAACAAGAGAGACCC |
| OAS2-F | CTCAGAAGCTGGGTTGGTTTAT |
| OAS2-R | ACCATCTCGTCGATCAGTGTC |
| PRRX1-F | TGATGCTTTTGTGCGAGAAGA |
| PRRX1-R | AGGGAAGCGTTTTTATTGGCT |
| PMEPA1-F | TGTCAGGCAACGGAATCCC |
| PMEPA1-R | CAGGTACGGATAGGTGGGC |
| GAPDH-F | GGTGAAGGTCGGAGTCAACG |
| GAPDH-R | CAAAGTTGTCATGGATGACC |
| si-SORBS2 | UCCGGAAUCCCCACAGCAATT |
| scramble-1 | ACCACUAACACCAGCCACGTT |
| si-MTUS1 | GGGUAAUCGAGGGCUUAAATT |
| scramble-2 | GUAGGGAGAGAAUAGUAGATT |
| 5’UTR-F | TAATACGACTCACTATAGGATCTCGTCGCTGTCACCTTGA |
| 5’UTR-R | ATGAGAGGGTGGGCAAAATGGTC |
| CDS-F | TAATACGACTCACTATAGGGCTCGTCATTTGGACTGACTT |
| CDS-R | GATCCTCTGAGGAGATACGGCTC |
| 3’UTR-F | TAATACGACTCACTATAGATTTCGGCATCGACACGGACGTTG |
| 3’UTR-R | CTTTTGTGGCTCTGGATGGTGGAG |
