# Supplementary material for: RNA-binding protein SORBS2 suppresses clear cell renal cell carcinoma metastasis by enhancing MTUS1 mRNA stability
Source: Cell Death Dis. 2020 Dec 12;11(12):1056. doi: 10.1038/s41419-020-03268-1 (PMC7732854; doi:10.1038/s41419-020-03268-1)
Supplement: Supplementary file 2 — Table S2 [file 41419_2020_3268_MOESM2_ESM.docx]

**Table S2. Clinicopathological features of patients with ccRCC downloaded from TCGA**

| **ID** | **Age** | **Sex** | **Stage** | **Survival** | **Days** | **FPKM** |
| --- | --- | --- | --- | --- | --- | --- |
| TCGA-A3-3306-01A | 67 | male | I | alive | 1120 | 5.5 |
| TCGA-A3-3307-01A | 66 | male | I | alive | 1436 | 7.6 |
| TCGA-A3-3308-01A | 77 | female | I | alive | 16 | 7 |
| TCGA-A3-3311-01A | 58 | male | Ⅲ | dead | 1191 | 4.1 |
| TCGA-A3-3313-01A | 59 | male | I | dead | 735 | 5.5 |
| TCGA-A3-3316-01A | 57 | male | Ⅲ | alive | 1493 | 8.7 |
| TCGA-A3-3317-01A | 67 | male | Ⅱ | alive | 1491 | 11.9 |
| TCGA-A3-3319-01A | 70 | male | IV | alive | 1130 | 1 |
| TCGA-A3-3320-01A | 52 | female | IV | alive | 1508 | 5.2 |
| TCGA-A3-3322-01A | 51 | male | IV | alive | 1478 | 9.5 |
| TCGA-A3-3323-01A | 53 | male | I | alive | 1106 | 6.4 |
| TCGA-A3-3324-01A | 51 | male | I | alive | 1186 | 6.9 |
| TCGA-A3-3325-01A | 52 | male | IV | dead | 1170 | 7.7 |
| TCGA-A3-3326-01A | 47 | male | I | alive | 1137 | 5.5 |
| TCGA-A3-3328-01A | 79 | male | Ⅲ | alive | 1385 | 1.6 |
| TCGA-A3-3329-01A | 75 | male | Ⅲ | alive | 1624 | 7.6 |
| TCGA-A3-3331-01A | 86 | female | Ⅲ | alive | 1485 | 8.8 |
| TCGA-A3-3335-01A | 41 | male | IV | alive | 1886 | 11.9 |
| TCGA-A3-3343-01A | 79 | male | IV | alive | 945 | 5.8 |
| TCGA-A3-3346-01A | 68 | male | IV | dead | 137 | 3.7 |
| TCGA-A3-3347-01A | 76 | female | IV | dead | 1610 | 6.3 |
| TCGA-A3-3349-01A | 34 | female | Ⅲ | alive | 1385 | 7.7 |
| TCGA-A3-3351-01A | 42 | male | I | alive | 910 | 7.2 |
| TCGA-A3-3357-01A | 62 | male | I | alive | 2688 | 7.3 |
| TCGA-A3-3358-01A | 57 | female | IV | alive | 1307 | 3.6 |
| TCGA-A3-3359-01A | 82 | female | I | alive | 2504 | 6.1 |
| TCGA-A3-3362-01A | 60 | female | IV | alive | 1559 | 6.3 |
| TCGA-A3-3363-01A | 50 | male | I | alive | 319 | 3.5 |
| TCGA-A3-3365-01A | 46 | male | I | alive | 873 | 12.1 |
| TCGA-A3-3367-01A | 72 | male | Ⅲ | alive | 2270 | 6.9 |
| TCGA-A3-3370-01A | 48 | female | IV | alive | 2274 | 5.4 |
| TCGA-A3-3372-01A | 64 | male | IV | alive | 735 | 8.8 |
| TCGA-A3-3373-01A | 54 | female | I | alive | 1621 | 5.3 |
| TCGA-A3-3374-01A | 51 | female | I | alive | 1314 | 3.1 |
| TCGA-A3-3376-01A | 51 | male | Ⅲ | dead | 1696 | 7.4 |
| TCGA-A3-3378-01A | 60 | male | IV | alive | 630 | 4.7 |
| TCGA-A3-3380-01A | 54 | male | IV | alive | 567 | 5.6 |
| TCGA-A3-3382-01A | 69 | male | IV | alive | 574 | 1.1 |
| TCGA-A3-3383-01A | 52 | male | IV | alive | 861 | 7.1 |
| TCGA-A3-3385-01A | 46 | female | IV | alive | 1993 | 7 |
| TCGA-A3-3387-01A | 49 | male | Ⅲ | alive | 617 | 4.8 |
| TCGA-A3-A6NI-01A | 47 | male | Ⅱ | alive | 1018 | 3.4 |
| TCGA-A3-A6NJ-01A | 57 | female | IV | alive | 468 | 4.3 |
| TCGA-A3-A6NL-01A | 49 | female | IV | alive | 689 | 4.9 |
| TCGA-A3-A6NN-01A | 78 | male | Ⅱ | alive | 3 | 8 |
| TCGA-A3-A8CQ-01A | 59 | female | Ⅲ | alive | 3 | 3.3 |
| TCGA-A3-A8OV-01A | 75 | male | IV | alive | 340 | 4.9 |
| TCGA-A3-A8OW-01A | 37 | male | IV | alive | 323 | 7.2 |
| TCGA-B0-4688-01A | 46 | male | IV | dead | 101 | 1.3 |
| TCGA-B0-4690-01A | 65 | male | IV | dead | 43 | 3 |
| TCGA-B0-4691-01A | 55 | male | I | dead | 139 | 3 |
| TCGA-B0-4693-01A | 73 | female | IV | dead | 77 | 5.3 |
| TCGA-B0-4694-01A | 72 | male | IV | dead | 106 | 6.1 |
| TCGA-B0-4696-01A | 58 | male | I | dead | 866 | 2.1 |
| TCGA-B0-4697-01A | 46 | female | Ⅲ | dead | 578 | 0.9 |
| TCGA-B0-4698-01A | 75 | male | I | dead | 42 | 0.1 |
| TCGA-B0-4699-01A | 74 | male | I | dead | 110 | 2.2 |
| TCGA-B0-4700-01A | 60 | male | I | dead | 1980 | 2.4 |
| TCGA-B0-4701-01A | 66 | female | I | dead | 238 | 3.5 |
| TCGA-B0-4703-01A | 51 | male | I | dead | 182 | 4.6 |
| TCGA-B0-4706-01A | 61 | male | I | dead | 65 | 7.2 |
| TCGA-B0-4707-01A | 63 | male | I | dead | 600 | 1.2 |
| TCGA-B0-4710-01A | 75 | female | Ⅱ | alive | 1755 | 5.8 |
| TCGA-B0-4712-01A | 76 | male | I | dead | 1337 | 2.6 |
| TCGA-B0-4713-01A | 76 | female | I | dead | 202 | 0.5 |
| TCGA-B0-4714-01A | 81 | male | IV | dead | 99 | 4.2 |
| TCGA-B0-4718-01A | 57 | male | I | alive | 1778 | 5.6 |
| TCGA-B0-4810-01A | 47 | male | I | dead | 478 | 4.2 |
| TCGA-B0-4811-01A | 48 | male | IV | dead | 1417 | 3 |
| TCGA-B0-4813-01A | 68 | male | Ⅲ | dead | 18 | 1.5 |
| TCGA-B0-4814-01A | 58 | male | IV | dead | 168 | 7.5 |
| TCGA-B0-4815-01A | 65 | male | Ⅲ | dead | 1588 | 1.2 |
| TCGA-B0-4816-01A | 49 | male | I | dead | 1371 | 5.5 |
| TCGA-B0-4817-01A | 81 | male | I | dead | 1019 | 2.2 |
| TCGA-B0-4818-01A | 68 | female | I | dead | 510 | 6.6 |
| TCGA-B0-4819-01A | 60 | female | I | dead | 183 | 3.8 |
| TCGA-B0-4821-01A | 68 | female | I | dead | 1230 | 1.1 |
| TCGA-B0-4822-01A | 78 | male | Ⅱ | dead | 1111 | 2.4 |
| TCGA-B0-4823-01A | 88 | male | IV | dead | 454 | 5.4 |
| TCGA-B0-4824-01A | 49 | female | IV | dead | 1657 | 4 |
| TCGA-B0-4827-01A | 77 | female | I | dead | 885 | 10 |
| TCGA-B0-4828-01A | 79 | male | I | dead | 307 | 6.3 |
| TCGA-B0-4833-01A | 82 | female | I | dead | 2386 | 4.7 |
| TCGA-B0-4834-01A | 49 | male | Ⅲ | dead | 2090 | 1.3 |
| TCGA-B0-4836-01A | 61 | male | I | dead | 1238 | 8.6 |
| TCGA-B0-4837-01A | 63 | male | IV | dead | 1378 | 4.9 |
| TCGA-B0-4838-01A | 69 | female | IV | dead | 834 | 5.8 |
| TCGA-B0-4839-01A | 80 | female | Ⅲ | dead | 1639 | 4.5 |
| TCGA-B0-4841-01A | 63 | male | Ⅲ | dead | 204 | 3.1 |
| TCGA-B0-4842-01A | 74 | female | Ⅲ | dead | 1724 | 0.3 |
| TCGA-B0-4843-01A | 57 | male | I | dead | 320 | 4.6 |
| TCGA-B0-4844-01A | 60 | male | I | dead | 313 | 4.4 |
| TCGA-B0-4845-01A | 70 | male | Ⅲ | dead | 1986 | 7.9 |
| TCGA-B0-4846-01A | 52 | male | Ⅲ | dead | 1200 | 6 |
| TCGA-B0-4847-01A | 60 | male | IV | dead | 793 | 2.5 |
| TCGA-B0-4848-01A | 54 | male | Ⅲ | dead | 883 | 8.1 |
| TCGA-B0-4849-01A | 51 | male | Ⅲ | dead | 69 | 5.1 |
| TCGA-B0-4852-01A | 78 | female | Ⅲ | dead | 1121 | 5.3 |
| TCGA-B0-4945-01A | 75 | female | I | dead | 2145 | 7.6 |
| TCGA-B0-5075-01A | 77 | female | I | dead | 637 | 4.3 |
| TCGA-B0-5077-01A | 77 | male | I | dead | 1317 | 4.1 |
| TCGA-B0-5080-01A | 63 | male | Ⅱ | dead | 342 | 3.8 |
| TCGA-B0-5081-01A | 80 | female | IV | dead | 362 | 3.3 |
| TCGA-B0-5083-01A | 63 | male | IV | dead | 1045 | 4.5 |
| TCGA-B0-5084-01A | 33 | male | I | dead | 222 | 1.8 |
| TCGA-B0-5085-01A | 76 | female | I | dead | 770 | 2.5 |
| TCGA-B0-5088-01A | 53 | male | Ⅲ | dead | 563 | 4.4 |
| TCGA-B0-5092-01A | 53 | female | Ⅲ | dead | 459 | 3 |
| TCGA-B0-5094-01A | 62 | male | IV | dead | 333 | 3 |
| TCGA-B0-5095-01A | 81 | male | I | dead | 245 | 5 |
| TCGA-B0-5096-01A | 72 | female | IV | dead | 68 | 0.4 |
| TCGA-B0-5097-01A | 59 | female | Ⅲ | alive | 665 | 2.9 |
| TCGA-B0-5098-01A | 53 | female | Ⅲ | dead | 1584 | 1.4 |
| TCGA-B0-5099-01A | 88 | female | I | dead | 485 | 6.8 |
| TCGA-B0-5100-01A | 72 | male | I | dead | 1913 | 8.3 |
| TCGA-B0-5102-01A | 74 | female | Ⅲ | dead | 2764 | 4.7 |
| TCGA-B0-5104-01A | 90 | female | Ⅲ | dead | 2752 | 4.4 |
| TCGA-B0-5106-01A | 64 | male | Ⅲ | dead | 1598 | 4.4 |
| TCGA-B0-5107-01A | 65 | female | IV | dead | 927 | 0.1 |
| TCGA-B0-5108-01A | 54 | male | I | alive | 1782 | 4 |
| TCGA-B0-5109-01A | 69 | male | IV | dead | 587 | 1.3 |
| TCGA-B0-5110-01A | 71 | female | Ⅱ | alive | 2009 | 7.2 |
| TCGA-B0-5113-01A | 69 | female | Ⅱ | alive | 1175 | 6.3 |
| TCGA-B0-5115-01A | 43 | male | Ⅲ | alive | 1604 | 7.8 |
| TCGA-B0-5116-01A | 53 | male | Ⅲ | alive | 1274 | 3.5 |
| TCGA-B0-5117-01A | 40 | male | Ⅲ | alive | 1608 | 2.8 |
| TCGA-B0-5119-01A | 61 | female | I | alive | 1552 | 10.2 |
| TCGA-B0-5120-01A | 72 | female | Ⅱ | alive | 1169 | 6.9 |
| TCGA-B0-5121-01A | 56 | male | I | alive | 1485 | 3.6 |
| TCGA-B0-5399-01A | 46 | male | I | alive | 1411 | 8 |
| TCGA-B0-5400-01A | 59 | female | I | alive | 1733 | 2.7 |
| TCGA-B0-5402-01A | 64 | male | Ⅲ | alive | 1290 | 14.3 |
| TCGA-B0-5690-01A | 53 | female | IV | alive | 3392 | 10.1 |
| TCGA-B0-5691-01A | 66 | female | I | alive | 3431 | 7.5 |
| TCGA-B0-5692-01A | 66 | female | I | alive | 3944 | 5.3 |
| TCGA-B0-5693-01A | 47 | female | I | alive | 4074 | 7.1 |
| TCGA-B0-5694-01A | 71 | male | Ⅲ | dead | 480 | 5.4 |
| TCGA-B0-5695-01A | 61 | female | Ⅱ | alive | 2150 | 7.8 |
| TCGA-B0-5696-01A | 70 | male | I | alive | 2609 | 7.2 |
| TCGA-B0-5697-01A | 50 | male | Ⅱ | alive | 2630 | 3.7 |
| TCGA-B0-5698-01A | 77 | male | I | alive | 3631 | 6.3 |
| TCGA-B0-5699-01A | 53 | male | I | alive | 3841 | 6.6 |
| TCGA-B0-5700-01A | 77 | male | I | alive | 1790 | 3.5 |
| TCGA-B0-5701-01A | 65 | male | Ⅲ | alive | 2461 | 3.9 |
| TCGA-B0-5702-01A | 71 | male | I | alive | 2172 | 2.4 |
| TCGA-B0-5703-01A | 73 | male | I | alive | 2246 | 7.9 |
| TCGA-B0-5705-01A | 65 | female | I | alive | 4537 | 6.3 |
| TCGA-B0-5706-01A | 45 | male | IV | alive | 3205 | 5.6 |
| TCGA-B0-5707-01A | 39 | female | Ⅲ | alive | 3744 | 3.2 |
| TCGA-B0-5709-01A | 62 | female | Ⅲ | alive | 3974 | 5.3 |
| TCGA-B0-5710-01A | 57 | male | Ⅲ | alive | 2430 | 13.4 |
| TCGA-B0-5711-01A | 50 | male | I | alive | 3989 | 10.7 |
| TCGA-B0-5712-01A | 68 | female | I | alive | 2722 | 5.7 |
| TCGA-B0-5713-01A | 75 | female | I | alive | 2782 | 7.2 |
| TCGA-B0-5812-01A | 53 | male | I | alive | 3834 | 11.8 |
| TCGA-B2-3923-01A | 59 | male | I | alive | 992 | 4.1 |
| TCGA-B2-3924-01A | 73 | male | IV | alive | 1092 | 4.2 |
| TCGA-B2-4098-01A | 72 | female | I | dead | 51 | 2.7 |
| TCGA-B2-4099-01A | 83 | male | Ⅱ | alive | 972 | 4.9 |
| TCGA-B2-4101-01A | 52 | male | Ⅲ | alive | 648 | 7.8 |
| TCGA-B2-4102-01A | 61 | male | Ⅱ | alive | 952 | 6.6 |
| TCGA-B2-5633-01B | 56 | male | Ⅲ | alive | 963 | 13.8 |
| TCGA-B2-5635-01A | 74 | male | I | alive | 755 | 4.4 |
| TCGA-B2-5636-01A | 79 | male | I | alive | 919 | 10.2 |
| TCGA-B2-5639-01A | 46 | male | I | dead | 1003 | 7.4 |
| TCGA-B2-5641-01A | 79 | male | I | alive | 656 | 6.7 |
| TCGA-B2-A4SR-01A | 62 | male | Ⅲ | alive | 507 | 17.8 |
| TCGA-B4-5377-01A | 68 | female | Ⅲ | alive | 365 | 6.7 |
| TCGA-B4-5378-01A | 62 | male | IV | alive | 175 | 7.4 |
| TCGA-B4-5832-01A | 65 | male | Ⅲ | alive | 155 | 8.7 |
| TCGA-B4-5834-01A | 59 | male | I | alive | 26 | 9.6 |
| TCGA-B4-5835-01A | 64 | female | Ⅲ | alive | 16 | 3.3 |
| TCGA-B4-5836-01A | 61 | female | Ⅲ | alive | 141 | 7.1 |
| TCGA-B4-5843-01A | 45 | male | I | alive | 11 | 11 |
| TCGA-B4-5844-01A | 61 | female | Ⅲ | alive | 7 | 13.5 |
| TCGA-B8-4143-01A | 66 | female | Ⅲ | dead | 709 | 0.2 |
| TCGA-B8-4146-01B | 41 | female | I | alive | 511 | 6.3 |
| TCGA-B8-4148-01A | 63 | female | IV | alive | 1520 | 7.5 |
| TCGA-B8-4151-01A | 51 | female | I | alive | 1299 | 7.3 |
| TCGA-B8-4153-01B | 74 | male | Ⅲ | alive | 762 | 6.8 |
| TCGA-B8-4154-01A | 73 | female | I | alive | 1380 | 5.9 |
| TCGA-B8-4619-01A | 58 | male | I | alive | 523 | 1.5 |
| TCGA-B8-4620-01A | 70 | female | I | alive | 777 | 2.3 |
| TCGA-B8-4621-01A | 63 | male | I | alive | 788 | 6.3 |
| TCGA-B8-4622-01A | 57 | male | I | alive | 1525 | 7.5 |
| TCGA-B8-5158-01A | 56 | male | IV | alive | 1218 | 6.5 |
| TCGA-B8-5159-01A | 61 | female | IV | alive | 722 | 8.9 |
| TCGA-B8-5162-01A | 63 | male | I | alive | 36 | 2.6 |
| TCGA-B8-5163-01A | 63 | female | I | alive | 822 | 1.9 |
| TCGA-B8-5164-01A | 65 | male | Ⅱ | alive | 26 | 5.6 |
| TCGA-B8-5165-01A | 43 | male | Ⅲ | alive | 737 | 9 |
| TCGA-B8-5545-01A | 42 | male | IV | alive | 1525 | 6.7 |
| TCGA-B8-5546-01A | 38 | female | Ⅲ | alive | 505 | 3.1 |
| TCGA-B8-5549-01A | 53 | male | I | alive | 194 | 4.4 |
| TCGA-B8-5550-01A | 71 | male | Ⅲ | alive | 1476 | 11.3 |
| TCGA-B8-5551-01A | 65 | female | Ⅲ | alive | 16 | 0.5 |
| TCGA-B8-5552-01B | 41 | female | Ⅲ | alive | 1046 | 8.3 |
| TCGA-B8-5553-01A | 67 | female | Ⅲ | alive | 435 | 5.7 |
| TCGA-B8-A54D-01A | 69 | male | Ⅱ | alive | 830 | 6.3 |
| TCGA-B8-A54E-01A | 62 | female | I | alive | 909 | 10.9 |
| TCGA-B8-A54F-01A | 49 | female | I | alive | 519 | 5.7 |
| TCGA-B8-A54G-01A | 50 | male | I | alive | 53 | 3.9 |
| TCGA-B8-A54H-01A | 69 | female | Ⅱ | alive | 256 | 5.7 |
| TCGA-B8-A54I-01A | 48 | male | Ⅲ | alive | 150 | 1.7 |
| TCGA-B8-A54J-01A | 60 | male | I | alive | 528 | 4.9 |
| TCGA-B8-A54K-01A | 61 | male | I | alive | 469 | 5.5 |
| TCGA-B8-A7U6-01A | 54 | female | Ⅱ | alive | 495 | 3.8 |
| TCGA-B8-A8YJ-01A | 60 | female | I | alive | 431 | 4.7 |
| TCGA-BP-4158-01A | 69 | male | IV | alive | 3377 | 6.4 |
| TCGA-BP-4159-01A | 70 | male | Ⅲ | dead | 2601 | 5.5 |
| TCGA-BP-4160-01A | 67 | male | Ⅲ | alive | 2881 | 5.1 |
| TCGA-BP-4161-01A | 74 | male | Ⅲ | alive | 2746 | 8 |
| TCGA-BP-4162-01A | 65 | female | I | alive | 3074 | 6.2 |
| TCGA-BP-4163-01A | 60 | female | Ⅱ | alive | 2839 | 7.3 |
| TCGA-BP-4164-01A | 51 | female | IV | dead | 992 | 6.8 |
| TCGA-BP-4165-01A | 64 | female | IV | alive | 3037 | 4 |
| TCGA-BP-4166-01A | 69 | male | Ⅲ | alive | 13 | 8 |
| TCGA-BP-4167-01A | 60 | male | I | alive | 2718 | 3.3 |
| TCGA-BP-4169-01A | 76 | female | I | dead | 701 | 5.2 |
| TCGA-BP-4170-01A | 72 | female | I | dead | 2343 | 4.7 |
| TCGA-BP-4173-01A | 47 | male | Ⅱ | alive | 1893 | 4.2 |
| TCGA-BP-4174-01A | 49 | male | I | alive | 1879 | 5.2 |
| TCGA-BP-4176-01A | 64 | male | Ⅱ | alive | 1955 | 7 |
| TCGA-BP-4177-01A | 65 | male | Ⅲ | alive | 1670 | 7.7 |
| TCGA-BP-4325-01A | 64 | female | I | alive | 2964 | 5.5 |
| TCGA-BP-4326-01A | 53 | female | I | dead | 1625 | 8.1 |
| TCGA-BP-4327-01A | 75 | female | Ⅲ | dead | 109 | 7.1 |
| TCGA-BP-4329-01A | 75 | male | IV | dead | 845 | 11.2 |
| TCGA-BP-4330-01A | 60 | female | I | alive | 1888 | 11.6 |
| TCGA-BP-4331-01A | 52 | male | Ⅲ | dead | 2454 | 4.7 |
| TCGA-BP-4332-01A | 36 | male | I | alive | 1133 | 10 |
| TCGA-BP-4334-01A | 56 | male | I | dead | 645 | 1.7 |
| TCGA-BP-4335-01A | 65 | female | I | dead | 475 | 5.5 |
| TCGA-BP-4337-01A | 76 | female | I | dead | 2 | 7.3 |
| TCGA-BP-4338-01A | 43 | male | I | alive | 2859 | 0.5 |
| TCGA-BP-4340-01A | 70 | female | I | dead | 562 | 5.9 |
| TCGA-BP-4341-01A | 67 | male | Ⅲ | dead | 1589 | 6.5 |
| TCGA-BP-4342-01A | 79 | male | Ⅲ | dead | 2256 | 10.9 |
| TCGA-BP-4343-01A | 64 | male | I | dead | 1912 | 3.6 |
| TCGA-BP-4344-01A | 75 | female | I | alive | 1666 | 6.2 |
| TCGA-BP-4345-01A | 62 | male | Ⅲ | alive | 1516 | 5.2 |
| TCGA-BP-4346-01A | 57 | male | IV | dead | 1493 | 5.3 |
| TCGA-BP-4347-01A | 74 | male | I | alive | 1367 | 5.5 |
| TCGA-BP-4349-01A | 68 | female | I | alive | 372 | 5.9 |
| TCGA-BP-4351-01A | 51 | female | Ⅱ | alive | 970 | 5.3 |
| TCGA-BP-4352-01A | 74 | female | I | dead | 344 | 0.1 |
| TCGA-BP-4353-01A | 61 | male | IV | dead | 375 | 7.3 |
| TCGA-BP-4354-01A | 41 | male | Ⅲ | dead | 1034 | 0.2 |
| TCGA-BP-4355-01A | 59 | female | I | dead | 953 | 6.5 |
| TCGA-BP-4756-01A | 63 | female | I | alive | 374 | 4 |
| TCGA-BP-4758-01A | 40 | male | Ⅱ | alive | 2208 | 3.9 |
| TCGA-BP-4759-01A | 50 | male | Ⅱ | alive | 2372 | 5.6 |
| TCGA-BP-4760-01A | 69 | male | Ⅲ | alive | 2361 | 6.1 |
| TCGA-BP-4761-01A | 57 | male | IV | alive | 182 | 0.2 |
| TCGA-BP-4762-01A | 42 | male | IV | dead | 1343 | 5.8 |
| TCGA-BP-4763-01A | 79 | female | I | dead | 1270 | 9.6 |
| TCGA-BP-4765-01A | 43 | male | Ⅱ | alive | 2184 | 7.1 |
| TCGA-BP-4766-01A | 43 | female | Ⅲ | alive | 1462 | 8 |
| TCGA-BP-4768-01A | 72 | female | Ⅲ | alive | 400 | 4.8 |
| TCGA-BP-4769-01A | 63 | male | I | alive | 1876 | 8.9 |
| TCGA-BP-4770-01A | 73 | female | Ⅲ | dead | 329 | 0.2 |
| TCGA-BP-4771-01A | 62 | male | Stage | dead | 162 | 2.9 |
| TCGA-BP-4774-01A | 57 | female | I | alive | 1885 | 5.6 |
| TCGA-BP-4775-01A | 55 | female | I | alive | 1843 | 13.1 |
| TCGA-BP-4776-01A | 52 | male | I | alive | 411 | 5.3 |
| TCGA-BP-4777-01A | 46 | male | Ⅲ | alive | 1731 | 4.6 |
| TCGA-BP-4781-01A | 78 | male | I | alive | 2080 | 6.4 |
| TCGA-BP-4782-01A | 55 | female | Ⅲ | alive | 354 | 6.6 |
| TCGA-BP-4784-01A | 67 | female | Ⅱ | alive | 1854 | 5.9 |
| TCGA-BP-4787-01A | 59 | female | IV | dead | 480 | 1.7 |
| TCGA-BP-4789-01A | 48 | male | IV | alive | 1489 | 8 |
| TCGA-BP-4790-01A | 76 | male | IV | dead | 1111 | 11.8 |
| TCGA-BP-4795-01A | 74 | female | I | alive | 620 | 8.3 |
| TCGA-BP-4797-01A | 34 | male | I | alive | 1107 | 8.7 |
| TCGA-BP-4798-01A | 74 | male | IV | dead | 334 | 1.2 |
| TCGA-BP-4799-01A | 70 | male | I | dead | 1133 | 1.2 |
| TCGA-BP-4801-01A | 57 | male | Ⅲ | alive | 1124 | 6.8 |
| TCGA-BP-4803-01A | 79 | male | Ⅲ | alive | 204 | 10.5 |
| TCGA-BP-4804-01A | 59 | male | Ⅲ | alive | 1459 | 6.5 |
| TCGA-BP-4807-01A | 42 | male | IV | alive | 211 | 8.1 |
| TCGA-BP-4959-01A | 49 | male | IV | alive | 2660 | 6.9 |
| TCGA-BP-4960-01A | 46 | male | IV | alive | 2172 | 4.2 |
| TCGA-BP-4961-01A | 47 | male | IV | alive | 1935 | 9.5 |
| TCGA-BP-4962-01A | 58 | male | Ⅲ | alive | 1785 | 8.2 |
| TCGA-BP-4963-01A | 64 | male | I | alive | 1834 | 8.6 |
| TCGA-BP-4964-01A | 55 | female | I | alive | 1862 | 5.6 |
| TCGA-BP-4965-01A | 46 | male | IV | alive | 1871 | 6.1 |
| TCGA-BP-4967-01A | 76 | male | I | alive | 205 | 8.2 |
| TCGA-BP-4968-01A | 40 | male | IV | alive | 1746 | 7.1 |
| TCGA-BP-4969-01A | 63 | female | I | alive | 1794 | 4.1 |
| TCGA-BP-4970-01A | 44 | male | I | alive | 433 | 6.8 |
| TCGA-BP-4971-01A | 40 | male | Ⅲ | alive | 1487 | 7.3 |
| TCGA-BP-4972-01A | 43 | female | IV | alive | 1502 | 8.9 |
| TCGA-BP-4973-01A | 47 | male | IV | alive | 1384 | 12.4 |
| TCGA-BP-4974-01A | 58 | male | I | dead | 211 | 6.2 |
| TCGA-BP-4975-01A | 40 | male | I | alive | 1433 | 7.6 |
| TCGA-BP-4976-01A | 77 | male | Ⅲ | alive | 1632 | 9.6 |
| TCGA-BP-4977-01A | 57 | male | IV | alive | 454 | 5 |
| TCGA-BP-4981-01A | 75 | female | IV | dead | 1097 | 3.5 |
| TCGA-BP-4982-01A | 42 | male | IV | alive | 1014 | 8.4 |
| TCGA-BP-4983-01A | 67 | female | IV | alive | 1413 | 0.5 |
| TCGA-BP-4985-01A | 72 | male | IV | dead | 952 | 1 |
| TCGA-BP-4986-01A | 75 | male | Ⅲ | alive | 785 | 5.7 |
| TCGA-BP-4987-01A | 41 | female | Ⅱ | alive | 1124 | 8.1 |
| TCGA-BP-4989-01A | 58 | male | IV | alive | 118 | 4.4 |
| TCGA-BP-4991-01A | 54 | male | IV | alive | 1413 | 10.1 |
| TCGA-BP-4992-01A | 66 | male | Ⅱ | alive | 501 | 1.5 |
| TCGA-BP-4993-01A | 58 | male | Ⅲ | alive | 177 | 4.9 |
| TCGA-BP-4994-01A | 54 | male | IV | alive | 1308 | 5.4 |
| TCGA-BP-4995-01A | 68 | male | IV | alive | 1371 | 6.2 |
| TCGA-BP-4998-01A | 49 | male | IV | alive | 932 | 7.4 |
| TCGA-BP-4999-01A | 56 | male | IV | alive | 1266 | 8.4 |
| TCGA-BP-5000-01A | 40 | male | I | alive | 563 | 5.1 |
| TCGA-BP-5001-01A | 43 | female | IV | alive | 1177 | 5.1 |
| TCGA-BP-5004-01A | 53 | male | IV | alive | 1126 | 8.8 |
| TCGA-BP-5006-01A | 61 | male | I | alive | 840 | 4.7 |
| TCGA-BP-5007-01A | 46 | male | Ⅲ | alive | 1140 | 8.1 |
| TCGA-BP-5008-01A | 46 | male | I | alive | 1071 | 8.1 |
| TCGA-BP-5009-01A | 52 | male | I | dead | 1092 | 5.4 |
| TCGA-BP-5010-01A | 63 | male | I | dead | 878 | 5.2 |
| TCGA-BP-5168-01A | 75 | male | I | dead | 1463 | 6.4 |
| TCGA-BP-5169-01A | 70 | male | I | alive | 193 | 3.8 |
| TCGA-BP-5170-01A | 55 | male | I | alive | 2412 | 8.8 |
| TCGA-BP-5173-01A | 75 | male | I | dead | 62 | 4.9 |
| TCGA-BP-5174-01A | 45 | female | Ⅱ | alive | 2257 | 5.5 |
| TCGA-BP-5175-01A | 60 | male | I | alive | 932 | 1.2 |
| TCGA-BP-5176-01A | 78 | female | I | dead | 1590 | 5.8 |
| TCGA-BP-5177-01A | 46 | female | IV | alive | 293 | 6.8 |
| TCGA-BP-5178-01A | 71 | male | I | dead | 1912 | 2.8 |
| TCGA-BP-5180-01A | 53 | male | I | alive | 2263 | 6.6 |
| TCGA-BP-5181-01A | 58 | female | IV | alive | 1495 | 10.9 |
| TCGA-BP-5182-01A | 56 | male | Ⅲ | alive | 1165 | 9.1 |
| TCGA-BP-5183-01A | 57 | male | IV | alive | 1291 | 4 |
| TCGA-BP-5184-01A | 54 | male | Ⅲ | alive | 1133 | 7.8 |
| TCGA-BP-5185-01A | 56 | male | I | alive | 1132 | 2 |
| TCGA-BP-5186-01A | 50 | female | I | alive | 693 | 5.5 |
| TCGA-BP-5187-01A | 54 | male | I | alive | 406 | 6.9 |
| TCGA-BP-5189-01A | 60 | male | I | dead | 822 | 7.5 |
| TCGA-BP-5190-01A | 61 | male | I | alive | 1011 | 5.7 |
| TCGA-BP-5191-01A | 80 | male | Ⅱ | alive | 967 | 1.8 |
| TCGA-BP-5192-01A | 59 | male | IV | alive | 714 | 6.2 |
| TCGA-BP-5194-01A | 39 | male | IV | alive | 408 | 7.6 |
| TCGA-BP-5195-01A | 75 | male | I | alive | 749 | 8.8 |
| TCGA-BP-5196-01A | 54 | male | I | alive | 1018 | 2.2 |
| TCGA-BP-5198-01A | 72 | male | I | alive | 603 | 5.7 |
| TCGA-BP-5199-01A | 58 | male | Ⅲ | alive | 1355 | 4.4 |
| TCGA-BP-5200-01A | 44 | male | I | alive | 1063 | 6.8 |
| TCGA-BP-5201-01A | 63 | male | IV | alive | 951 | 6.3 |
| TCGA-BP-5202-01A | 75 | male | IV | alive | 29 | 7.4 |
| TCGA-CJ-4634-01A | 60 | female | Ⅲ | alive | 3498 | 9.2 |
| TCGA-CJ-4635-01A | 48 | male | Ⅲ | alive | 1416 | 14.1 |
| TCGA-CJ-4636-01A | 51 | male | Ⅲ | alive | 1924 | 4.1 |
| TCGA-CJ-4637-01A | 52 | female | I | dead | 2227 | 2.1 |
| TCGA-CJ-4638-01A | 46 | female | I | dead | 431 | 8 |
| TCGA-CJ-4639-01A | 49 | female | Ⅲ | alive | 3229 | 6.9 |
| TCGA-CJ-4640-01A | 49 | male | Ⅲ | alive | 3480 | 5.8 |
| TCGA-CJ-4641-01A | 55 | female | IV | dead | 1661 | 6.3 |
| TCGA-CJ-4642-01B | 47 | male | Ⅲ | alive | 3205 | 5.8 |
| TCGA-CJ-4643-01A | 67 | female | Ⅲ | alive | 1793 | 7.5 |
| TCGA-CJ-4644-01A | 48 | female | Ⅲ | dead | 336 | 6.7 |
| TCGA-CJ-4868-01A | 42 | male | I | dead | 646 | 3.5 |
| TCGA-CJ-4869-01A | 49 | male | I | alive | 2554 | 4.8 |
| TCGA-CJ-4870-01A | 58 | female | I | alive | 1498 | 3.6 |
| TCGA-CJ-4871-01A | 63 | male | Ⅱ | alive | 2423 | 8.7 |
| TCGA-CJ-4872-01A | 51 | male | IV | alive | 326 | 9.2 |
| TCGA-CJ-4873-01A | 85 | female | IV | alive | 2259 | 3.8 |
| TCGA-CJ-4874-01A | 73 | female | I | alive | 2283 | 5.9 |
| TCGA-CJ-4875-01A | 67 | male | I | dead | 3554 | 4.6 |
| TCGA-CJ-4876-01A | 57 | male | Ⅲ | alive | 1955 | 3.9 |
| TCGA-CJ-4878-01A | 71 | female | Ⅲ | alive | 2186 | 4.6 |
| TCGA-CJ-4881-01A | 41 | male | IV | alive | 2014 | 2.3 |
| TCGA-CJ-4882-01A | 57 | male | I | alive | 1883 | 5.2 |
| TCGA-CJ-4884-01A | 72 | female | IV | alive | 1759 | 9.3 |
| TCGA-CJ-4885-01A | 64 | male | Ⅲ | alive | 3451 | 7.5 |
| TCGA-CJ-4886-01A | 43 | female | Ⅲ | alive | 1952 | 8.1 |
| TCGA-CJ-4887-01A | 48 | male | I | dead | 932 | 5.7 |
| TCGA-CJ-4888-01A | 59 | male | I | dead | 1567 | 2.7 |
| TCGA-CJ-4889-01A | 63 | female | Ⅲ | alive | 1946 | 5.4 |
| TCGA-CJ-4890-01A | 72 | male | Ⅲ | alive | 3519 | 3.4 |
| TCGA-CJ-4891-01A | 57 | female | Ⅲ | dead | 819 | 0.6 |
| TCGA-CJ-4892-01A | 65 | female | IV | alive | 1521 | 7.4 |
| TCGA-CJ-4893-01A | 76 | female | I | alive | 750 | 7.2 |
| TCGA-CJ-4894-01A | 58 | male | IV | dead | 841 | 5.7 |
| TCGA-CJ-4895-01A | 62 | male | Ⅱ | dead | 1200 | 4.8 |
| TCGA-CJ-4897-01A | 79 | female | Ⅱ | alive | 3341 | 9.3 |
| TCGA-CJ-4899-01A | 42 | male | Ⅲ | alive | 1528 | 7.7 |
| TCGA-CJ-4900-01A | 69 | female | Ⅲ | dead | 1714 | 1.6 |
| TCGA-CJ-4901-01A | 47 | male | Ⅲ | alive | 1450 | 4.4 |
| TCGA-CJ-4902-01A | 61 | male | I | alive | 1520 | 4.7 |
| TCGA-CJ-4903-01A | 50 | male | Ⅱ | alive | 1559 | 5.4 |
| TCGA-CJ-4904-01A | 60 | female | I | alive | 3302 | 7.1 |
| TCGA-CJ-4905-01A | 62 | female | I | alive | 1496 | 9.1 |
| TCGA-CJ-4907-01A | 58 | male | I | alive | 1499 | 10.4 |
| TCGA-CJ-4908-01A | 38 | male | Ⅲ | alive | 1531 | 6.7 |
| TCGA-CJ-4912-01A | 61 | male | IV | alive | 1657 | 4.7 |
| TCGA-CJ-4916-01A | 69 | female | I | alive | 1373 | 3.7 |
| TCGA-CJ-4918-01A | 64 | male | I | dead | 93 | 3.5 |
| TCGA-CJ-4920-01A | 64 | female | I | dead | 139 | 6 |
| TCGA-CJ-5671-01A | 51 | male | Ⅲ | alive | 3987 | 5.2 |
| TCGA-CJ-5672-01A | 84 | male | Ⅱ | dead | 2190 | 4.2 |
| TCGA-CJ-5675-01A | 70 | male | I | alive | 3936 | 5.1 |
| TCGA-CJ-5676-01A | 47 | male | Ⅱ | alive | 4067 | 5.7 |
| TCGA-CJ-5677-01A | 54 | female | I | dead | 782 | 0.6 |
| TCGA-CJ-5678-01A | 62 | male | I | dead | 574 | 2.6 |
| TCGA-CJ-5679-01A | 73 | male | I | dead | 679 | 5.4 |
| TCGA-CJ-5680-01A | 65 | female | Ⅲ | dead | 768 | 12.8 |
| TCGA-CJ-5681-01A | 44 | female | I | dead | 552 | 4.1 |
| TCGA-CJ-5682-01A | 60 | male | I | alive | 3736 | 7.4 |
| TCGA-CJ-5683-01A | 78 | male | I | alive | 1889 | 4.4 |
| TCGA-CJ-5684-01A | 61 | male | IV | alive | 2231 | 6.7 |
| TCGA-CJ-5686-01A | 59 | female | Ⅲ | alive | 2038 | 4.9 |
| TCGA-CJ-5689-01A | 90 | male | Ⅲ | dead | 1620 | 5 |
| TCGA-CJ-6027-01A | 77 | male | Ⅲ | dead | 3615 | 6.3 |
| TCGA-CJ-6028-01A | 58 | male | I | dead | 1625 | 4.2 |
| TCGA-CJ-6030-01A | 66 | male | I | dead | 2299 | 4.5 |
| TCGA-CJ-6031-01A | 54 | male | I | alive | 1906 | 4.1 |
| TCGA-CJ-6032-01A | 63 | female | I | alive | 3639 | 6.6 |
| TCGA-CJ-6033-01A | 54 | female | I | dead | 224 | 3.7 |
| TCGA-CW-5580-01A | 73 | female | IV | dead | 1964 | 2.9 |
| TCGA-CW-5581-01A | 44 | male | I | alive | 2799 | 10.6 |
| TCGA-CW-5583-01A | 51 | female | Ⅱ | alive | 2489 | 10.3 |
| TCGA-CW-5584-01A | 74 | male | Ⅲ | dead | 164 | 7.2 |
| TCGA-CW-5585-01A | 51 | male | Ⅱ | alive | 2609 | 10.4 |
| TCGA-CW-5587-01A | 62 | female | Ⅲ | alive | 2226 | 5.3 |
| TCGA-CW-5588-01A | 78 | female | I | alive | 2017 | 6.9 |
| TCGA-CW-5589-01A | 52 | male | I | alive | 2378 | 13.4 |
| TCGA-CW-5590-01A | 51 | male | I | dead | 1075 | 8.3 |
| TCGA-CW-5591-01A | 56 | male | I | alive | 2271 | 8.3 |
| TCGA-CW-6087-01A | 61 | male | Ⅲ | dead | 41 | 0.9 |
| TCGA-CW-6088-01A | 60 | male | Ⅲ | alive | 3222 | 14.4 |
| TCGA-CW-6090-01A | 68 | male | IV | alive | 2552 | 6 |
| TCGA-CW-6093-01A | 73 | male | Ⅲ | alive | 3146 | 6.7 |
| TCGA-CW-6097-01A | 32 | male | I | dead | 571 | 4.1 |
| TCGA-CZ-4853-01A | 82 | male | Ⅲ | alive | 774 | 8.6 |
| TCGA-CZ-4854-01A | 68 | male | Ⅲ | dead | 1404 | 3.8 |
| TCGA-CZ-4856-01A | 62 | female | I | alive | 18 | 5.2 |
| TCGA-CZ-4857-01A | 56 | male | Ⅲ | dead | 1432 | 4.2 |
| TCGA-CZ-4858-01A | 39 | male | Ⅲ | dead | 2105 | 0.1 |
| TCGA-CZ-4859-01A | 59 | female | I | alive | 1787 | 9 |
| TCGA-CZ-4860-01A | 60 | male | IV | dead | 206 | 1 |
| TCGA-CZ-4861-01A | 63 | male | I | dead | 446 | 2.3 |
| TCGA-CZ-4862-01A | 46 | male | Ⅲ | alive | 3271 | 6.5 |
| TCGA-CZ-4863-01A | 51 | female | I | alive | 1928 | 4 |
| TCGA-CZ-4864-01A | 86 | male | I | dead | 2830 | 3.8 |
| TCGA-CZ-4865-01A | 70 | female | I | dead | 166 | 3.8 |
| TCGA-CZ-4866-01A | 79 | female | I | alive | 3267 | 12.3 |
| TCGA-CZ-5451-01A | 74 | male | I | alive | 1929 | 9.5 |
| TCGA-CZ-5452-01A | 69 | male | IV | alive | 1789 | 5.6 |
| TCGA-CZ-5453-01A | 67 | male | IV | dead | 2419 | 7.9 |
| TCGA-CZ-5454-01A | 63 | male | I | dead | 722 | 5.6 |
| TCGA-CZ-5455-01A | 63 | male | I | dead | 561 | 5.4 |
| TCGA-CZ-5456-01A | 57 | male | Ⅱ | alive | 2422 | 8.6 |
| TCGA-CZ-5457-01A | 62 | male | Ⅲ | alive | 2754 | 6.2 |
| TCGA-CZ-5458-01A | 43 | male | IV | alive | 2789 | 6.3 |
| TCGA-CZ-5459-01A | 63 | male | Ⅲ | alive | 1683 | 3.9 |
| TCGA-CZ-5460-01A | 55 | male | I | alive | 2873 | 5.1 |
| TCGA-CZ-5461-01A | 52 | male | Ⅲ | dead | 330 | 5.5 |
| TCGA-CZ-5462-01A | 83 | male | Ⅲ | dead | 311 | 3.3 |
| TCGA-CZ-5463-01A | 76 | male | Ⅲ | alive | 662 | 7.7 |
| TCGA-CZ-5464-01A | 69 | male | Ⅲ | alive | 2128 | 6 |
| TCGA-CZ-5465-01A | 76 | female | Ⅱ | dead | 2564 | 8 |
| TCGA-CZ-5466-01A | 67 | male | I | alive | 685 | 4.7 |
| TCGA-CZ-5467-01A | 86 | female | I | dead | 73 | 6.2 |
| TCGA-CZ-5468-01A | 84 | male | I | dead | 59 | 0.6 |
| TCGA-CZ-5469-01A | 41 | male | Ⅱ | dead | 946 | 2.5 |
| TCGA-CZ-5470-01A | 72 | female | Ⅲ | alive | 386 | 7.1 |
| TCGA-CZ-5982-01A | 59 | female | I | alive | 2439 | 6.5 |
| TCGA-CZ-5984-01A | 51 | male | I | alive | 2067 | 3.5 |
| TCGA-CZ-5985-01A | 58 | male | Ⅱ | alive | 1997 | 6.9 |
| TCGA-CZ-5986-01A | 61 | male | I | alive | 373 | 14 |
| TCGA-CZ-5987-01A | 60 | male | IV | dead | 445 | 6.9 |
| TCGA-CZ-5988-01A | 38 | male | Ⅲ | alive | 693 | 3.9 |
| TCGA-CZ-5989-01A | 60 | male | Ⅲ | alive | 1905 | 11.2 |
| TCGA-DV-5565-01A | 59 | male | Ⅲ | alive | 1329 | 3.2 |
| TCGA-DV-5566-01A | 67 | female | I | alive | 1398 | 5.2 |
| TCGA-DV-5567-01A | 40 | female | Ⅱ | alive | 2004 | 9 |
| TCGA-DV-5568-01A | 26 | male | IV | alive | 370 | 5.8 |
| TCGA-DV-5569-01A | 29 | female | IV | alive | 355 | 3.3 |
| TCGA-DV-5573-01A | 41 | male | Ⅲ | alive | 1130 | 5.2 |
| TCGA-DV-5574-01A | 37 | male | I | alive | 2016 | 7.1 |
| TCGA-DV-5575-01A | 52 | female | I | alive | 1729 | 8.8 |
| TCGA-DV-5576-01A | 55 | female | I | dead | 727 | 9.1 |
| TCGA-DV-A4VX-01A | 59 | male | Ⅱ | dead | 1626 | 2.7 |
| TCGA-DV-A4VZ-01A | 53 | male | I | alive | 365 | 1.7 |
| TCGA-DV-A4W0-01A | 55 | male | Ⅱ | alive | 2470 | 6.6 |
| TCGA-EU-5904-01A | 47 | female | Ⅲ | alive | 551 | 9.3 |
| TCGA-EU-5905-01A | 67 | female | I | alive | 119 | 3.7 |
| TCGA-EU-5906-01A | 55 | male | I | alive | 206 | 7.9 |
| TCGA-EU-5907-01A | 81 | male | Ⅲ | alive | 127 | 6 |
