# Supplementary material for: RNA-binding protein SORBS2 suppresses clear cell renal cell carcinoma metastasis by enhancing MTUS1 mRNA stability
Source: Cell Death Dis. 2020 Dec 12;11(12):1056. doi: 10.1038/s41419-020-03268-1 (PMC7732854; doi:10.1038/s41419-020-03268-1)
Supplement: Supplementary file 3 — Table S3 [file 41419_2020_3268_MOESM3_ESM.docx]

**Table S3. Potential targeted genes of SORBS2**

| **Gene_Symbol** | **logFC** | **adj.P.Val** |
| --- | --- | --- |
| MTUS1 | 7.24 | 0.02001 |
| CD83 | 5.79 | 0.01713 |
| ZDBF2 | 4.43 | 0.01406 |
| IL17D | 4.21 | 0.00907 |
| MAPK4 | 3.68 | 0.02459 |
| SNAI1 | 3.46 | 0.00446 |
| ING3 | 3.29 | 0.00057 |
| FBXO25 | 3.02 | 0.01808 |
| HOXB8 | 2.43 | 0.01964 |
| BMPE | 2.35 | 0.00073 |
| PRRG2 | 2.14 | 0.00791 |
| NEBL | 2.00 | 0.00537 |
| NDUFA4L2 | -2.07 | 0.02014 |
| TAP1 | -2.19 | 0.02791 |
| SYBU | -2.20 | 0.02425 |
| CDH11 | -2.21 | 0.02684 |
| PMEPA1 | -2.28 | 0.01701 |
| COL1A1 | -2.29 | 0.02661 |
| ISG15 | -2.41 | 0.02677 |
| PRRX1 | -2.46 | 0.02980 |
| THBS2 | -2.77 | 0.00990 |
| COL6A3 | -2.88 | 0.01476 |
| BIRC3 | -2.98 | 0.01447 |
| PDGFRB | -3.08 | 0.00638 |
| POSTN | -3.39 | 0.02929 |
| TMEM119 | -3.51 | 0.02988 |
| ALDH1A3 | -3.56 | 0.02395 |
| OAS2 | -4.29 | 0.00962 |
| PLOD2 | -4.55 | 0.00114 |
