# Supplementary figures and images for: RNA-binding protein SORBS2 suppresses clear cell renal cell carcinoma metastasis by enhancing MTUS1 mRNA stability
Source: Cell Death Dis. 2020 Dec 12;11(12):1056. doi: 10.1038/s41419-020-03268-1 (PMC7732854; doi:10.1038/s41419-020-03268-1)

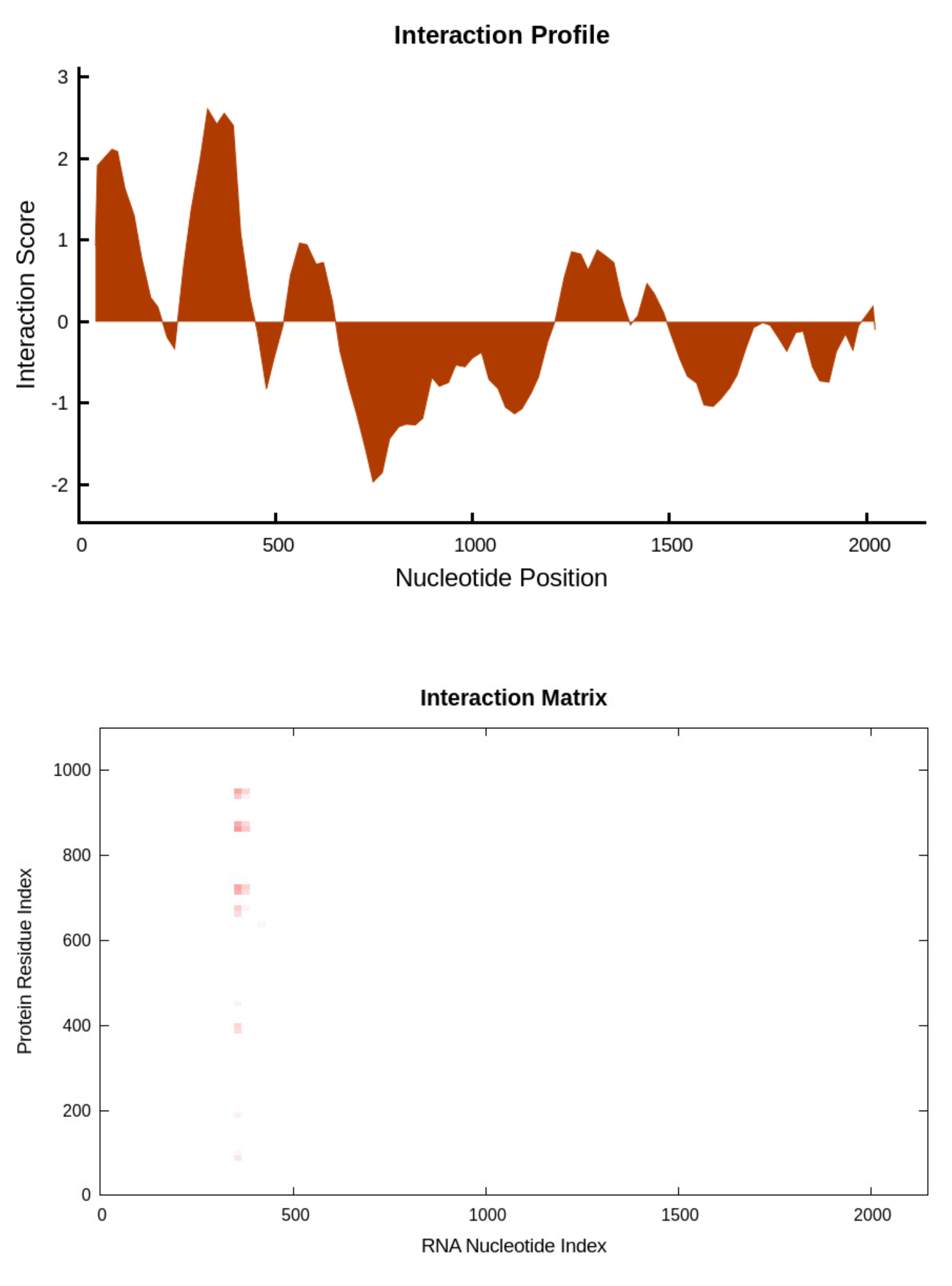

Supplement: Supplementary file 4 — Figure S1 [file 41419_2020_3268_MOESM4_ESM.tif]

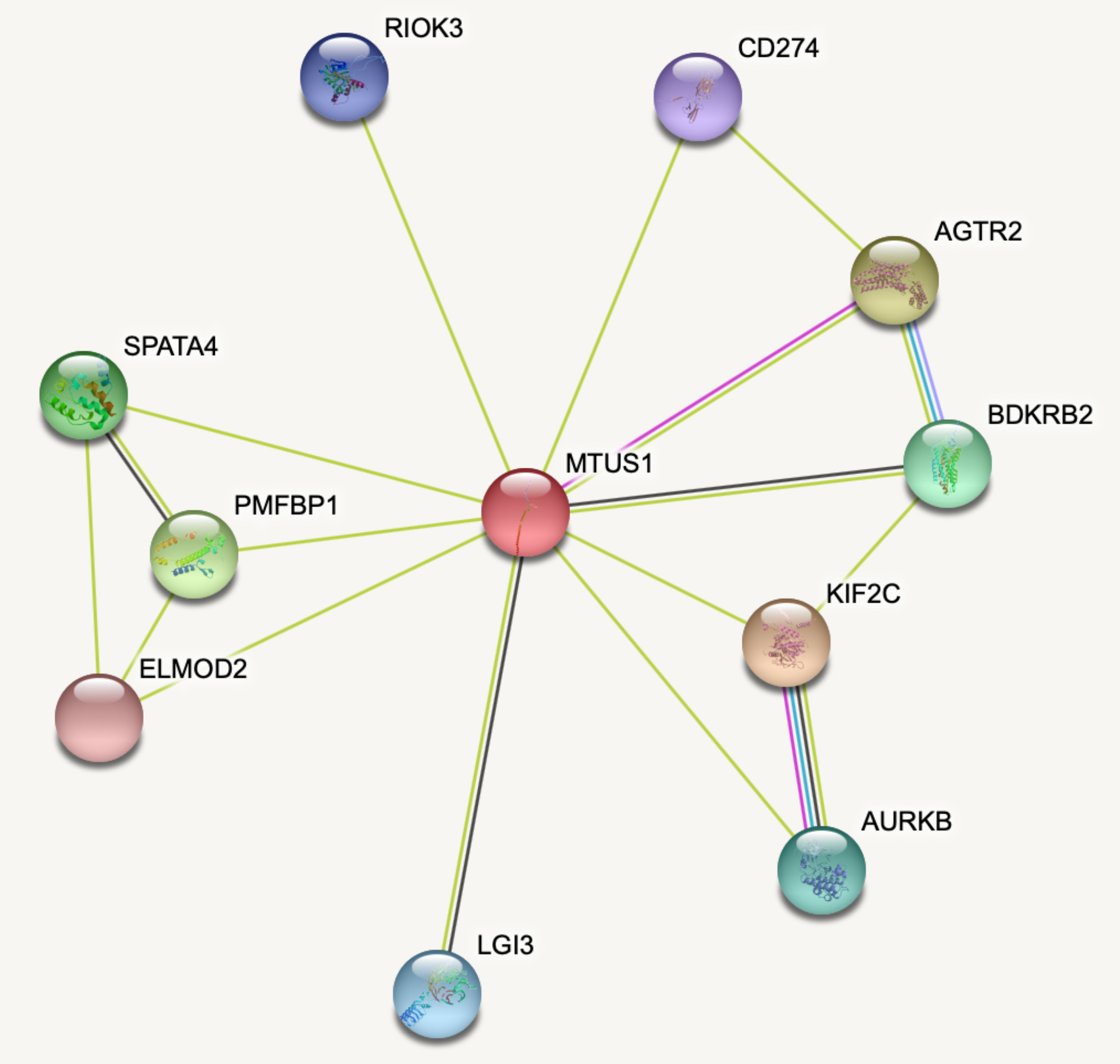

Supplement: Supplementary file 5 — Figure S2 [file 41419_2020_3268_MOESM5_ESM.tif]
