# Supplementary material for: RNA-binding protein SORBS2 suppresses clear cell renal cell carcinoma metastasis by enhancing MTUS1 mRNA stability
Source: Cell Death Dis. 2020 Dec 12;11(12):1056. doi: 10.1038/s41419-020-03268-1 (PMC7732854; doi:10.1038/s41419-020-03268-1)
Supplement: Supplementary file 6 — Supplementary Figure Legends [file 41419_2020_3268_MOESM6_ESM.docx]

**Supplementary Figure Legends**

**Fig. S1 catRAPID predicates the binding domain between SORBS2 protein and MTUS1 mRNA.**

**Fig. S2 Predication of potential molecules associated with MTUS1 by STING database.**
